# Deciphering how Cpl-7 cell wall-binding repeats recognize the bacterial peptidoglycan

**DOI:** 10.1038/s41598-017-16392-4

**Published:** 2017-11-28

**Authors:** Noemí Bustamante, Manuel Iglesias-Bexiga, Noelia Bernardo-García, Noella Silva-Martín, Guadalupe García, María A. Campanero-Rhodes, Esther García, Isabel Usón, Rubén M. Buey, Pedro García, Juan A. Hermoso, Marta Bruix, Margarita Menéndez

**Affiliations:** 1 Instituto de Química-Física Rocasolano, Consejo Superior de Investigaciones Científicas, Serrano 119, 28006 Madrid, Spain; 2 CIBER de Enfermedades Respiratorias (CIBERES), Madrid, Spain; 3 Centro de Investigaciones Biológicas, Consejo Superior de Investigaciones Científicas, Ramiro de Maeztu 9, 28040 Madrid, Spain; 4 Instituto de Biología Molecular de Barcelona, CSIC, Baldiri Reixach 13, 08028 Barcelona, Spain; 5 ICREA (Institució Catalana de Recerca i Estudis Avançats), Barcelona, Spain; 6 Metabolic Engineering Group. Departamento de Microbiología y Genética, Universidad de Salamanca, Campus Miguel de Unamuno, 37007 Salamanca, Spain

## Abstract

Endolysins, the cell wall lytic enzymes encoded by bacteriophages to release the phage progeny, are among the top alternatives to fight against multiresistant pathogenic bacteria; one of the current biggest challenges to global health. Their narrow range of susceptible bacteria relies, primarily, on targeting specific cell-wall receptors through specialized modules. The cell wall-binding domain of Cpl-7 endolysin, made of three CW_7 repeats, accounts for its extended-range of substrates. Using as model system the cell wall-binding domain of Cpl-7, here we describe the molecular basis for the bacterial cell wall recognition by the CW_7 motif, which is widely represented in sequences of cell wall hydrolases. We report the crystal and solution structure of the full-length domain, identify *N*-acetyl-D-glucosaminyl-(β1,4)-*N*-acetylmuramyl-L-alanyl-D-isoglutamine (GMDP) as the peptidoglycan (PG) target recognized by the CW_7 motifs, and characterize feasible GMDP-CW_7 contacts. Our data suggest that Cpl-7 cell wall-binding domain might simultaneously bind to three PG chains, and also highlight the potential use of CW_7-containing lysins as novel anti-infectives.

## Introduction

Antibiotic-resistant bacteria are especially promiscuous and constitute an increasing source of healthcare and economic concerns. Repeated warnings raised on the possibility of a post-antibiotic era have emphasized the need of developing new treatments to ensure a sustainable control of many diseases, whose causal agents include human and animal pathogens as well as food spoilage bacteria. Lysins, the cell wall lytic enzymes encoded by bacteriophages (endolysins) or bacteria (autolysins), have attracted much attention because of their ability to break-down the cell wall of target bacteria when added exogenously (lysis-from-without)^1,2^. This novel class of antibacterials has important advantages over classical antibiotics, *e*.*g*., a novel mode of action; a narrow spectrum of susceptible bacteria; rapid killing of both stationary- and exponentially-growing bacteria; activity on mucous membranes and bacterial biofilms; low probability of developing resistances; and reduced impact on normal microbiota^3–5^. These unique features have boosted the interest on the biotechnological and pharmacological exploitation of lysins^1,6–8^ and their recent inclusion among the top ten current alternatives to fight antibiotic resistances^9^.

Lysins from Gram-positive bacteria and their phages usually comprise at least one catalytic domain and one or more cell wall-binding domains^5,6,10–12^. In contrast, many lysins produced by Gram-negative species or their phages only contain the catalytic domain^5,12^, though modular endolysins have also been reported^13–15^. The catalytic units dictate the type of peptidoglycan (PG) bond to be cleaved, whereas the cell wall-binding domain(s) largely determines the lytic spectrum by specific recognition of cell wall elements distributed in genus-, or species/strain-specific manner^5,10,12^. Other factors helping to define bacterial susceptibility are the net charge of lysin domains, the fine architecture of the bacterial envelope and the overall three-dimensional structure of the lysin^16–18^.

**Figure 1:**
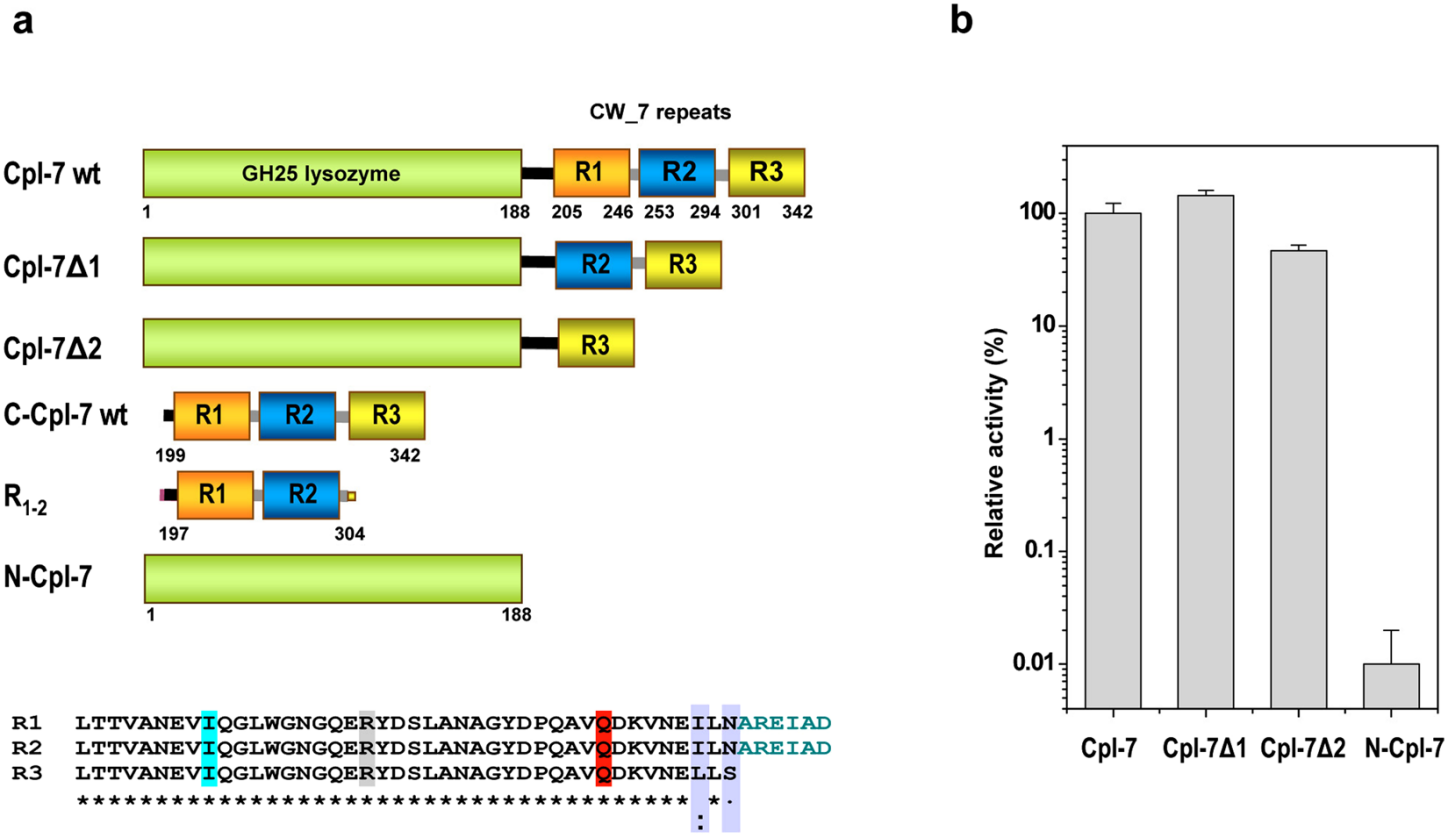
Module composition of Cpl-7 endolysin, deletion constructs and contribution of CW_7 repeats to the murolytic activity. (**a**) Cartoon representation of Cpl-7 variants used in this study. Numbers indicate residues comprised in each structural element. The catalytic domain, in green, belongs to the GH-25 family. The cell wall-binding domain comprises three almost identical CW_7 repeats of 42 residues (R1, R2 and R3; sequence alignment is shown at the bottom) connected by six-residue-long inter-repeat linkers (AREIAD). The residues substituted by alanine in C-Cpl-7R, C-Cpl-7Q and C-Cpl-7I mutants are highlighted in grey, red and cyan, respectively. (**b**) Variation of the percentage of specific activity on pneumococcal cell walls with serial deletion of the CW_7 repeats from Cpl-7 sequence. Full-length Cpl-7 specific activity was taken as 100%. Data are the average of three independent assays of four-to-six replicas each; bars indicated standard deviations.

The Cpl-7 endolysin encoded by the pneumococcal Cp-7 bacteriophage comprises a catalytic domain with muramidase activity and a cell wall-binding domain made up of three almost identical repeats (CW_7 repeats hereafter) (Fig. 1a). Because of CW_7 repeats, Cpl-7 can hydrolyze pneumococcal cell walls containing either choline or ethanolamine^19^, and its lytic spectrum is extended to streptococcal (*Streptococcus pneumoniae*, *Streptococcus pyogenes*, *Streptococcus mitis* and *Streptococcus dysgalactiae*) and non-streptococcal pathogens (*Enterococcus faecalis*) compared with other pneumococcal lysins^17,18^. Moreover, in the presence of destabilizing agents of the outer membrane, Cpl-7 and its improved variant, Cpl-7S, can also kill Gram-negative bacteria^17^. Of note, CW_7 repeats are present in many putative cell wall hydrolases encoded by Gram-positive and Gram-negative bacteria as well as by (pro)phages of Gram-positive bacteria^11,12^ and they consist of 37–43 amino acids predicted to adopt a three-helix bundle fold^11^. To date, only two other CW_7 comprising enzymes have been biochemically characterized and their bacteriolytic activity demonstrated: the endolysins encoded by the λSa2 prophage of *Streptococcus agalactiae* strain 2603 v/r^20^ and by the SMP bacteriophage of *Streptococcus suis* ^21^. However, the variety of organisms coding for CW_7-containing proteins strongly suggest that CW_7 repeats would likely target a conserved element of the bacterial cell wall.

Here, we have employed a multidisciplinary approach to positively identify the element of the bacterial cell wall recognized by the CW_7 repeats and to outline the lysin/cell-wall interactions mediated by them, using as a model the multimodular cell wall binding domain of Cpl-7 (C-Cpl-7). By means of saturation transfer difference NMR spectroscopy (STD-NMR) we have demonstrated that GMDP is recognized as a ligand by the CW_7 repeats, and the binding epitope of this fragment of the PG monomer has been identified. The crystal structure of one single repeat and the complete C-Cpl-7 domain have been elucidated and the latter compared with the domain structure in solution, which was investigated by small angle X-ray scattering (SAXS). Moreover, a docking model of the C-Cpl-7 domain in complex with GMDP consistent with the STD-NMR mapping and mutational studies is proposed. In addition, we have explored how the number of repeats and their sequence variability might affect lysin activity and specificity. Taken together, our results shed light on the fine interactions established between key amino acid residues of CW_7 repeats and the building blocks of the PG, providing a rational for the use of this cell wall-binding motif in lysin design and applications.

## Results

### CW_7 motifs in nature

**Figure 2:**
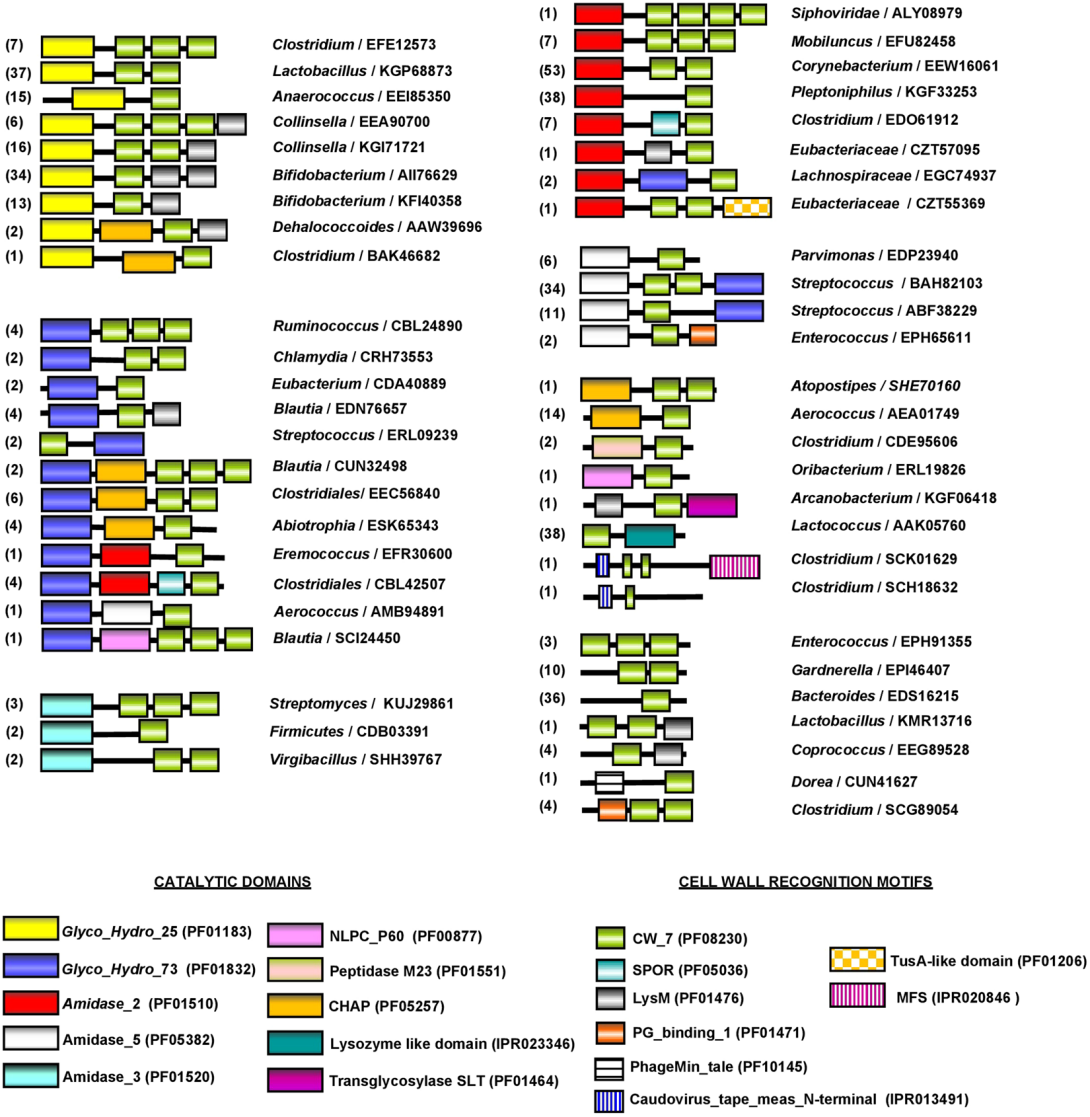
Schematic representation of protein architectures containing the CW_7 motif. The accession number of representative proteins and the respective coding bacterium are indicated. Figures in parenthesis are the number of sequences within each architecture. Comprised modules are colour coded and Pfam or INTERPRO accession codes are shown at the bottom.

Analysis of protein data bases (Interpro IPR013168 and Pfam PF08230 accession codes) showed that CW_7 motifs are present in a great variety of bacterial genes (455; last accession date 8 May, 2017), organized in highly different non-redundant architectures (Fig. 2). Functional domains carried by most of them suggest their implication in cell wall metabolism or bacterial lysis. The majority of the bacterial species belong to the Actinobacteria (127) and Firmicutes (293) phila, with predominance of Actinomycetaceae (30) and Bifidobacteriaceae (67) families in Actinobacteria, and of Streptococcaceae (88) and Lachnospiraceae (55) in Firmicutes (http://www.ebi.ac.uk/interpro/entry/IPR013168/taxonomy). Most of them are normal components of human and cattle epithelial microbioma, although beneficial and pathogenic species are also present, as well as opportunistic pathogens. The catalytic domains of the most frequent architectures belong to families GH25, Amidase_2, Lysozyme like domain (IPR023346), and to Amidase_5 in combination with glucosaminidase GH73 (Fig. 2). Allocation of respective architectures to bacterial species made evident their uneven distribution between Eubacteria and Firmicutes phyla (Supplementary Fig. S1). So, the Amidase_5-(CW_7)_1/2_-GH73 architectures appear only in streptococci, and the CW_7-Lysozyme like domain in lactococci. Besides, GH25-(CW_7)_1/2_ and GH25-(CW_7)_2_-LysM sequences are more frequent in Firmicutes than in Actinobacteria. Contrarily, the GH25-(CW_7)-LysM is favoured in Actinobacteria, and further acquisition of an extra LysM domain in GH25-(CW_7)-LysM_2_ reduced its presence to the *Bifidobacterium* genus. Additionally, Amidase_2-(CW_7) and Amidase_2-(CW_7)_2_ architectures predominate in Firmicutes or Actinobacteria, respectively. On the other hand, the CW_7 repeats from Firmicutes are generally three residues longer than those from Actinobacteria (Supplementary Figs S2 and S3). However, repeats of GH25-(CW_7)_2_-LysM and Amidase_5-(CW_7)_2_-GH73 architectures are almost all short, and those of GH25-(CW_7)_2_ long, independently of their origin. Long and short repeats coexist in a few Amidase_2-(CW_7)_2_ sequences (Supplementary Fig. S3a). In addition to this, comparison of the intra-repeat connecting linkers revealed the existence of common traits within specific architectures (like, for example, high proline content in Amidase_2-(CW_7)_2/3_ sequences but not in GH25-(CW_7)_2/3_). However, sequence and length may even differ between linkers connecting consecutive CW_7 repeats (Supplementary Fig. S3). Taken together, all these observations support the notion that, in addition to functional domains, evolutionary sequence variation has extended to the non-conserved regions of the linkers that, presumably, play some structural role as well.

### Effect of Cpl-7 repeats on lytic activity

The high number of proteins putatively involved in cell wall metabolism carrying just one CW_7 motif strongly indicates that a single repeat may recognize its binding target on the bacterial surface, either alone or helped by other cell wall-binding domains. To test this hypothesis, DNA fragments encoding constructs comprising the catalytic module of Cpl-7 fused to the last (Cpl-7Δ2) or the two last (Cpl-7Δ1) CW_7 repeats (Fig. 1a; Supplementary Fig. S4) were cloned, and the truncated proteins overexpressed and purified. Ultracentrifugation experiments revealed a positive correlation between the sedimentation coefficient, or the Stokes’ radii, and the number of repeats in the constructs (Supplementary Table S1). Besides, the far- and near-UV circular dichroism (CD) spectra indicated that they were properly folded (Supplementary Fig. S5). When comparing their specific activities on purified pneumococcal cell walls with that of the separated catalytic domain (N-Cpl-7), an increase of three-to four orders of magnitude was found for Cpl-7Δ1 and Cpl-7Δ2 constructs. Notably, Cpl-7Δ1, with only two repeats, appeared to be even more active (≈ 40%) than the full-length enzyme (Fig. 1b).

### Crystal structure of the CW_7 repeat and the full-length C-Cpl-7 domain

**Figure 3:**
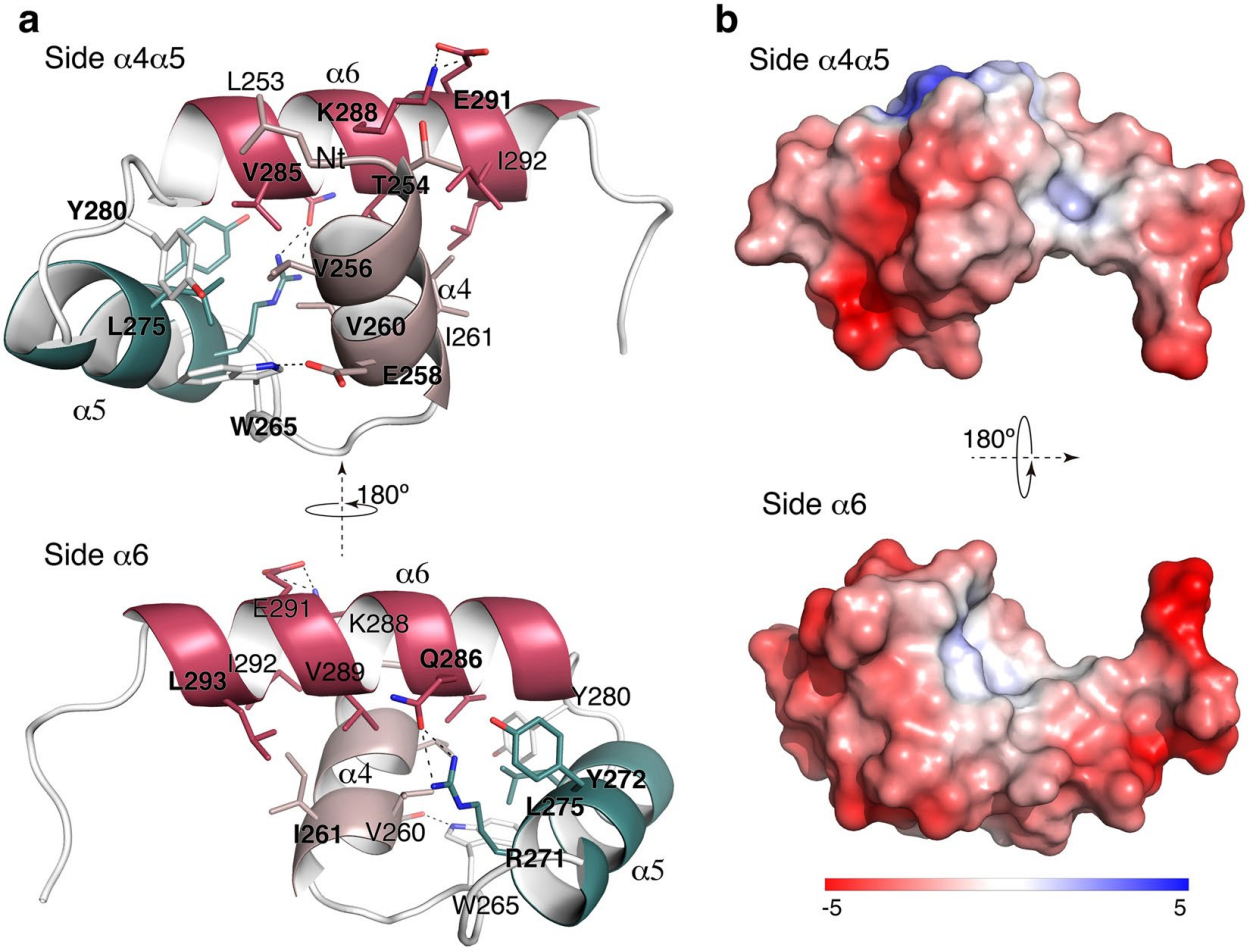
Crystal structure of the CW_7 repeat of Cpl-7. (**a**) Ribbon representation of the R2 repeat showing views of the side α4α5 (top) and the side α6 (bottom). Relevant residues are depicted as capped-sticks. A hydrophobic patch is observed at the core of the three-helix bundle. Hydrogen bonds and salt-bridge interactions are displayed with dot lines. (**b**) Poisson-Boltzmann electrostatic-potential surface (color bar range ± 5 kT/e) generated by PyMOL APBS tool^79^ for side α4α5 (top) and side α6 (bottom) of the R2 repeat.

The three CW_7 repeats of Cpl-7 endolysin (R1, R2, and R3 hereafter), made of 42 amino acids each, are connected by six-residue-long linkers (Fig. 1a). Aiming to investigate the molecular mechanism underpinning their interaction with the bacterial cell wall, we undertook the crystallisation of the C-Cpl-7 domain. Crystals were first grown in 1.3 M trisodium citrate using the wild type domain (C-Cpl-7 wt) isolated from the LSLt-C-Cpl-7 fusion protein (see Methods). They diffracted at high-resolution (1.6 Å) and contained just one repeat (referred to as R2 hereafter) at the asymmetric unit (see Supplementary Methods). Solving the R2 three-dimensional structure *de novo* using *ARCIMBOLDO* ^22^ (Supplementary Fig. S6) showed that it is composed of a bundle of three α helices stabilised by hydrophobic interactions (Fig. 3). The first two helices (α4 and α5 in the full-length domain numbering), both nine residues long, are antiparallel, and the longer α6 helix (13 amino acids) lies in diagonal on the two other helices (Fig. 3a). The resultant bundle presents two faces, one formed by helices α4 and α5 (side α4α5) and the other by the helix α6 (side α6). The repeat contains a hydrophobic core centred around Leu275 (located in the middle of helix α5), which is surrounded by Val256 and Val260 (helix α4), Val285 (helix α6) and Tyr280 (α5–α6 connecting loop) (Fig. 3a). Several polar interactions contribute also to the bundle’s stability. A strong hydrogen bond is formed between Arg271 and Gln286 that maintains the close conformation of helix α6 and helix α5. A salt-bridge interaction is also observed between Lys288 and Glu291 (both in helix α6). Besides, aromatic residues are capping both sides of the repeat (Tyr280 and Trp265 cap the α4α5 side and Tyr272 the α6 side). Of note, the repeat presents a strong acidic character in both sides (Fig. 3b). There is no close structural homologue of the CW_7 repeat after a search in DALI server^23^.

**Figure 4:**
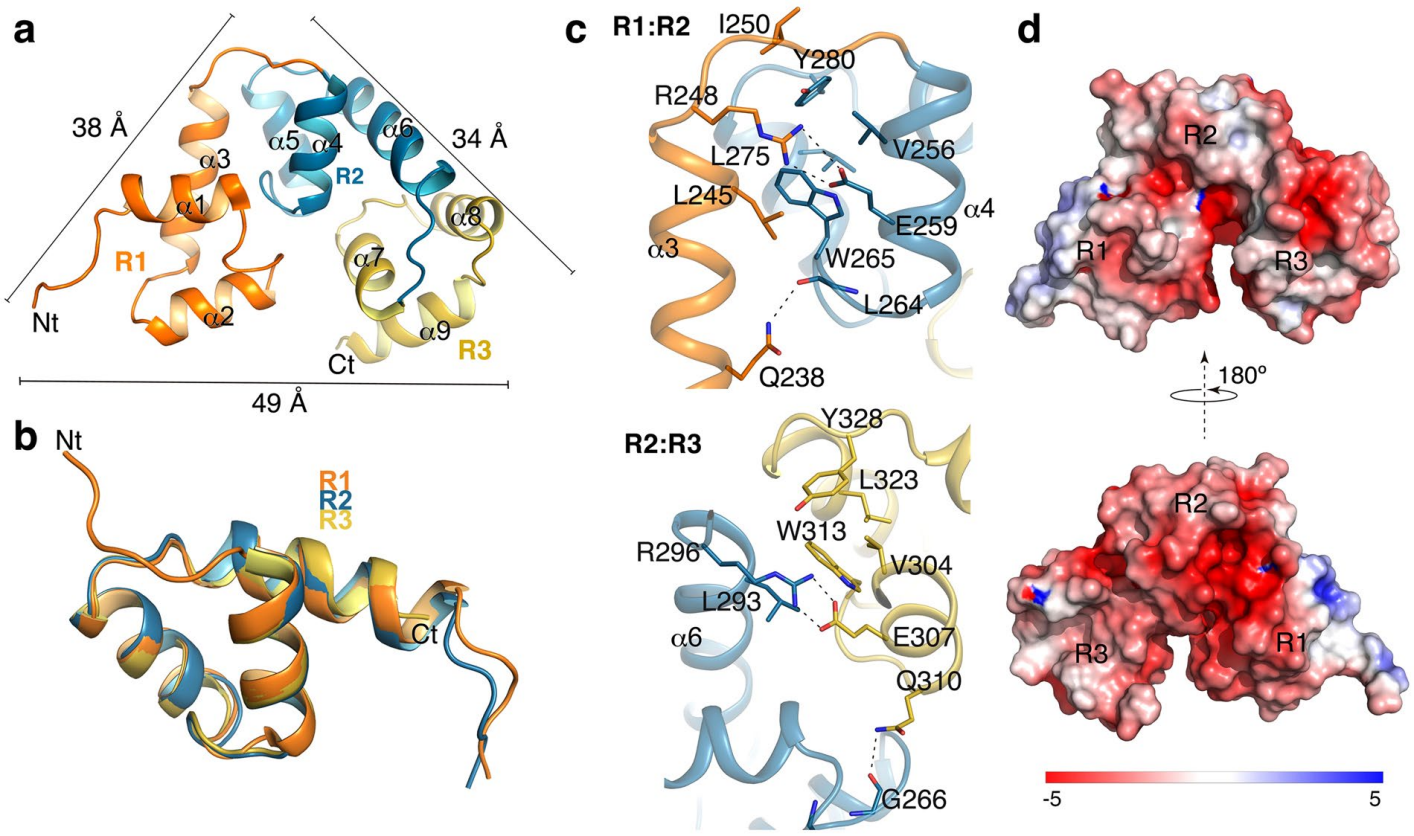
Structure of full-length C-Cpl-7R domain. (**a**) Crystal structure of C-Cpl-7R with repeats coloured in orange (R1), blue (R2) and yellow (R3). Comprised helices are labelled from α1 to α9. Bars indicate the side dimensions of the C-Cpl-7R triangular structure in angstroms. (**b**) Structural superimposition of the three repeats of C-Cpl-7R. (**c**) Interactions between repeats R1 and R2 repeats (top panel) and R2 and R3 repeats (bottom panel) coloured as in (**a**). Residues involved in inter-repeat interactions are depicted as sticks, and polar interactions as dotted lines. (**d**) Poisson-Boltzmann electrostatic-potential surface (color bar range ± 5 kT/e) generated by PyMOL APBS tool^79^ for the full-length C-Cpl-7R domain.

In a further effort to solve the three-dimensional structure of the full-length domain, DNA fragments encoding C-Cpl-7 wt or a triple arginine-to-alanine mutant (C-Cpl-7R) at positions 223 (R1), 271(R2) and 319 (R3) (fully conserved in the sequences of the CW_7 family and likely involved in cell wall recognition; see below) were cloned and purified without the LSLt tag (see Methods). Crystals that diffracted up to 2.8 Å resolution were obtained only for C-Cpl-7R in 1.1 M trisodium citrate. The crystal structure of C-Cpl-7R was solved by the molecular replacement method using the 3D structure of R2 repeat as initial model. It showed a tightly closed and packed conformation (dimensions of 49 × 38 × 34 Å) of the three repeats that followed a pseudo-three-fold symmetry (Fig. 4a). In agreement with almost full conservation of the sequences of the repeats (Fig. 1), the three bundles present nearly identical structures (RMSD of 0.62 Å and 0.49 Å for Cα atoms of R2 and R3 over R1, respectively) with minor differences in the inter-repeat linkers (Fig. 4b). R1 has an N-terminal extension, due to the presence of the six last residues from the linker (see Supplementary Fig. S4), and slightly differs from R2 and R3 also in the structure of the loop that joins helices α1 and α2. In addition, the lack of the inter-repeat linker at the end of R3 shortens the helix α9 (Fig. 4b). The overall C-Cpl-7R structure is stabilized by polar and electrostatic contacts between consecutive repeats (Fig. 4c). The central interaction at the R1:R2 interface is a salt-bridge between Arg248 (R1-to-R2 loop) and Glu259 (R2) side chains. Other contacts of special relevance are the hydrogen bond established between Gln238 (R1) and the main chain of Leu264 (R2), and the cation-π interaction between Trp265 (R2) and Arg248 (R1). Similar interactions were observed at the R2:R3 interface (Fig. 4c). Once again, no significantly similar structures were found using the DALI server^23^ for the full-length domain.

Superimposition of the R2 repeats from C-Cpl-7R and the wild type form showed highly similar structures (RMSD of 0.31 Å for Cα atoms; Supplementary Fig. S7a). The substitution of arginine 271 by alanine disrupts the cation-π interaction established with Tyr272, whose side chain rotates in C-Cpl7- R to make a hydrogen bond with Q286, thereby helping to maintain the close conformation of the two last helices found in the wild type repeat. The same happened in the other two repeats. The small impact of the arginine substitution in the repeat structure, together with its inner location and the substantial distance between it and the nearest interface/s between repeats, support the notion that C-Cpl-7R and C-Cpl-7 wt may adopt similar overall conformations. Indeed, the small differences found between their near-CD spectra would be accounted for the tyrosine rotation (Supplementary Fig. S7c), and neither the far-UV CD spectrum nor the domain stability were considerably affected by the triple mutation (Supplementary Fig. S7b,d). The same happened with their hydrodynamic parameters, based on the sedimentation coefficients (*s*_20,w_), Stokes’ radii (*R*_s_) and frictional ratios (*f*/*f*_o_) determined by sedimentation velocity for C-Cpl-7 wt and C-Cpl-7R (Table 1). Therefore, our results also indicate that the full conservation of this arginine entails a functional role, rather than being essential for the bundle structure preservation.

**Table 1:** Hydrodynamic parameters of C-Cpl-7 wt and C-Cpl-7R determined by sedimentation velocity or SAXS compared with those calculated from the X-ray structure or the SAXS-derived models.

Hydrodynamic parameters	Sedimentation velocity^a^	Crystal structure^c^ (C-Cpl-7m)	SAXS data	BUNCH model^c^
*R*_*g*_ (Å) (from Guinier)	—	16.5	22.7	22.2
*R*_*g*_ (Å) (from *P*(r))	—	—	22.9	—
D_max_ (Å)	—	51	70	72
*s*_*20*,*w*_ (S)	1.6 (1.5)	1.8	—	1.6
*f/f*_*o*_ friction coefficient	1.46 (1.54)	1.2	—	1.4
*R*_*s*_ at 293 K (Å)	24.5 (25.3)	21.5	—	25
Estimated molecular mass (kDa)	16.2 (14.1)	—	17.2^**b**^	—
Molecular mass from sequence (kDa)		15.9 (15.6)		

^**a**^Values in parenthesis are for C-Cpl-7R. ^**b**^Calculated from the Porod’s volume. ^**c**^The hydrodynamic parameters for C-Cpl-7R crystal structure and the Bunch model were calculated from their atomic coordinates using WinHydropro.

### Determination of C-Cpl-7 conformation in solution by SAXS

**Figure 5:**
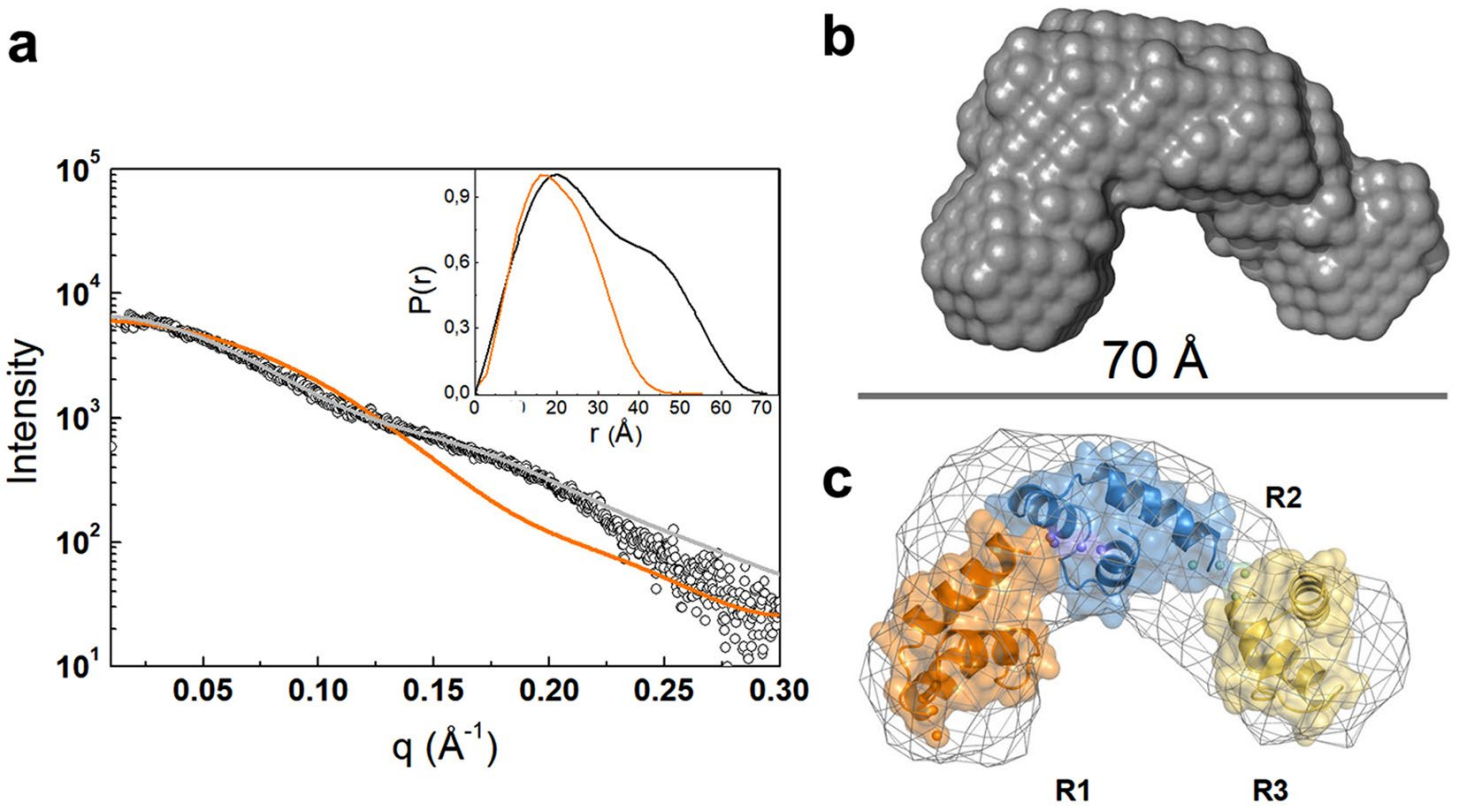
Determination of C-Cpl-7 configuration in solution by SAXS. (**a**) Overlay of the scattering curve of C-Cpl-7 wt at infinite dilution (black dots) with the scattering profile calculated from the full-length domain crystal structure using CRYSOL and the fit obtained by rigid-body modelling (BUNCH) (orange and grey traces, respectively). The inset shows the real-space distance-distribution functions *P*(r) for the configuration in solution (black trace) and the crystal structure (orange trace). (**b**) *Ab initio* bead model of C-Cpl-7 reconstructed from SAXS data using DAMMIF. (**c**) Superposition of the DAMMIF model in mesh representation with the best rigid model (cartoon) produced by BUNCH.

The hydrodynamic parameters computed from the atomic coordinates of the overall domain were perceptibly different from those measured in PB buffer for either C-Cpl-7 wt or C-Cpl-7R (Table 1), using sedimentation velocity. Based on the *s*_20,w_ and *R*_s_ values, the solution structure of both C-Cpl-7 variants appeared to be less compact than that of the crystal, suggesting that C-Cpl-7 conformation might depend on the surrounding medium. Thus, we performed SAXS experiments to determine the domain size and shape in solution. The experimental scattering profile largely departed from the SAXS spectrum calculated from the crystal atomic coordinates by CRYSOL^24^, and was consistent with that of a monomeric, non-globular particle (Fig. 5a and Table 1). Whereas the configuration in solution has a maximum intra-particle distance, *D*_max_, of 70 Å and a distance distribution function, *P*(r), with two maxima at 20 and 45 Å, the crystal structure has a *D*_max_ of 51 Å and a bell-shaped *P*(r) function with a maximum at 17 Å. The *ab initio* reconstruction of the C-Cpl-7 envelope with DAMMIF^25^ resulted in a boomerang-shaped model (Fig. 5b) that was notably less compact than the crystal structure. The *ab initio* model superposed well onto the rigid-body model reconstructed with BUNCH^26^ (Fig. 5c), which moved and rotated the repeat crystal structures as rigid bodies, treating the intra-repeat linkers as chains of dummy residues, to get the best fit of the scattering profile. The *R*_g_, *D*_max_, and particle volume of the BUNCH model compared well with the corresponding SAXS estimates, and its hydrodynamic parameters (*s*_20,w_, *f*/*f*_0_ and *R*_s_) matched those measured by sedimentation velocity (Table 1).

Additionally, the ensemble optimization method (EOM) analysis was employed for assessing the domain flexibility in solution using an initial random pool of 10,000 configurations^27^. As shown in Supplementary Fig. S8b, the ensemble of configurations selected by EOM was consistent with the presence of a single predominant conformation at the assayed conditions. Besides, the *R*_g_ average value of the selected configurations agreed with the experimental one and those of DAMMIF and BUNCH models, thereby confirming that they did not represent an average of highly distinct (opened or highly packed) configurations of C-Cpl-7 in solution. Nonetheless, EOM analysis was indicative of a certain rotational mobility of the repeats, illustrated by the superimposition of the three more populated conformations selected by EOM (Supplementary Fig. S8b,c). Thus, our SAXS analysis further supported the notion that C-Cpl-7 may adopt different conformations under different conditions, such as those employed for the crystallisation (1.1 M tri-sodium citrate) or the solution studies (20 mM phosphate PB buffer).

### Characterization of GMDP binding to C-Cpl-7 by STD-NMR

Thermal stabilization of C-Cpl-7 structure upon addition of GMDP^28^ prompted us to validate this analogue of the peptidoglycan monomer as binder of CW_7 repeats, and to map its binding epitope by using STD-NMR. This method is based on the transfer of magnetization from the protein, which is selectively irradiated by a train of radiofrequency pulses, to any binding ligand in fast exchange^29^. In small proteins like C-Cpl-7 (144 residues) magnetization transfer is less efficient, weakening STD effects, and, occasionally, different results are obtained from saturation of protons located in different protein regions^30^. Thus, STD experiments were performed irradiating either the aliphatic (-1 ppm) or the aromatic (7.7 ppm) regions of the protein. Conservation of domain native structure under NMR conditions was previously checked (see Methods and Supplementary Fig. S9).

**Figure 6:**
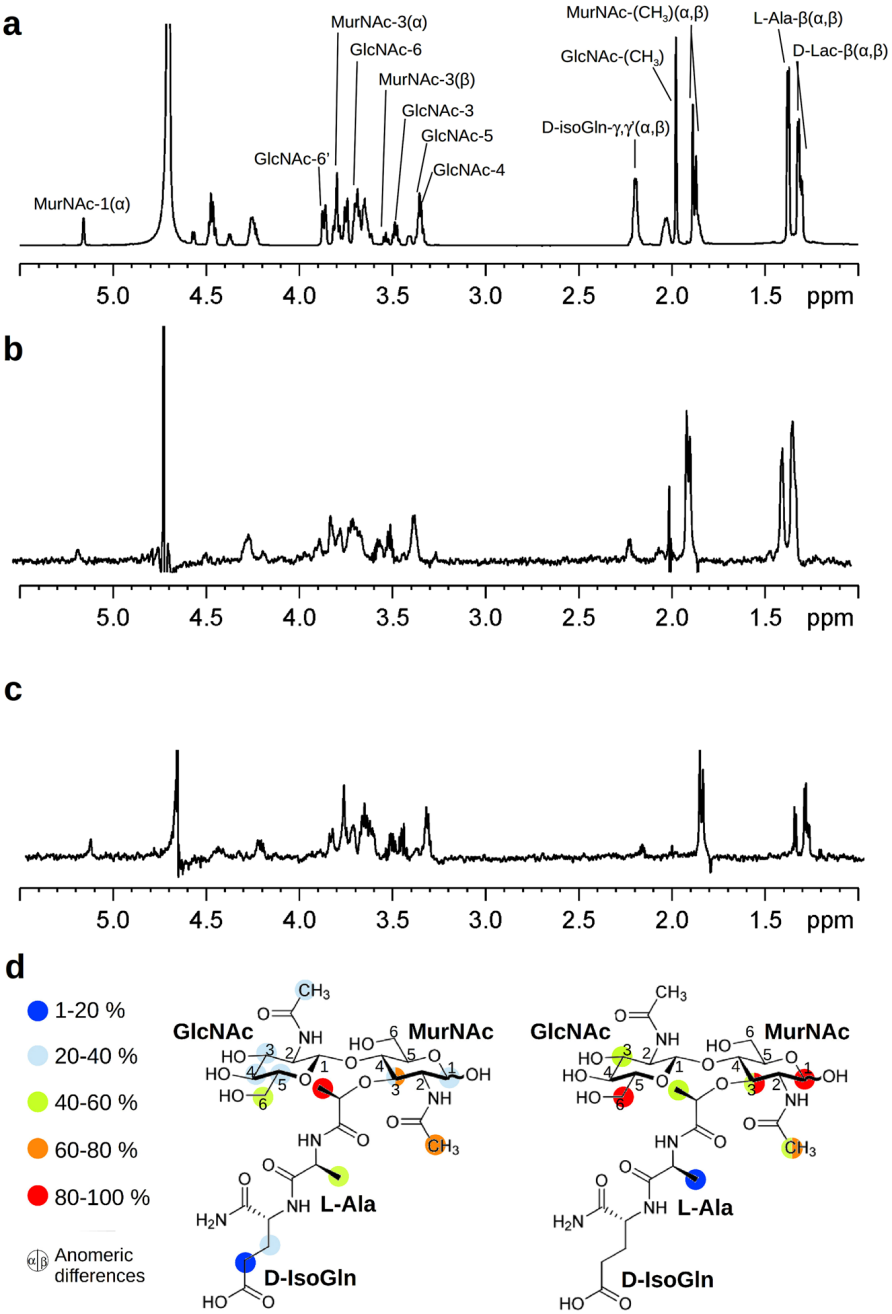
Characterization of GMDP binding to C-Cpl-7 by STD-NMR in PB buffer. (**a**) ^1^H NMR spectrum of free GMDP as reference. Proton accounting for relevant peaks are marked as in Supplementary Table S2 where chemical shifts of ^1^H NMR spectra of GMDP α:β anomer mixture (2:1 ratio) in D_2_O are shown. (**b**) and (**c**) STD-NMR spectra of GMDP (2.5 mM) in the presence of C-Cpl-7 wt (33 μM) upon irradiation at the methyl or the aromatic region, respectively. The 2:1 ratio between the α and β anomers of free GMDP changes to 1.3:1 in STD spectra, unveiling the preferential binding of the β anomer. (**d**) GMDP binding epitopes as deduced from STD data upon irradiation at methyl (left) or aromatic (right) protein regions. Colour labels indicate the STD intensity (I) of each signal relative to the most intense signal observed. Red circles: 100% > I > 80%; orange: 80% > I > 60%; green: 60% > I > 40%; cyan: 40% > I > 20%; blue: 20% > I > 1%.

Measurements carried out in the presence of C-Cpl-7 wt at the low ionic strength of solution studies (PB-D_2_O buffer) gave rise to STD-positive peaks (Fig. 6a–c), confirming GMDP binding. The signals were weak (less than 1.5% ligand intensities), as expected from the small protein size. STD spectra showed that the *β* anomer of GMDP (the one present in bacterial PG) was preferentially bound over the α anomer (1.3:1 ratio), and that GMDP interacts with C-Cpl-7 wt through the two saccharide units and the stem peptide. When the aliphatic groups were pulsed, the highest degree of saturation was observed on the signals of lactyl and *N*-acetyl methyl protons of MurNAc. However, upon irradiation of the aromatic signals, the strongest STD effects were seen at the signals of protons H6 of GlcNAc, H1 and H3 of MurNAc, and the *N*-acetyl methyl of MurNAc. The GMDP binding epitopes identified from each condition are summarized in Fig. 6d.

Further STD experiments were performed to investigate the effect of high salt concentration (to approach crystallisation conditions) on the domain binding capacity. In that setting (0.25 M sodium pyrophosphate, 1.5 M KCl, pD = 8.6), the STD signals of GMDP were extremely weak, and they were only detectable in the spectrum obtained from methyl irradiation (Supplementary Fig. S10). This result indicated that the muropeptide recognition could be largely mediated by electrostatic interactions or, alternatively, that the domain conformation and/or the binding site accessibility was ionic-strength dependent, which would be in line with the crystal structure and SAXS data.

### Modelling of GMDP in complex with CW_7 repeats

**Figure 7:**
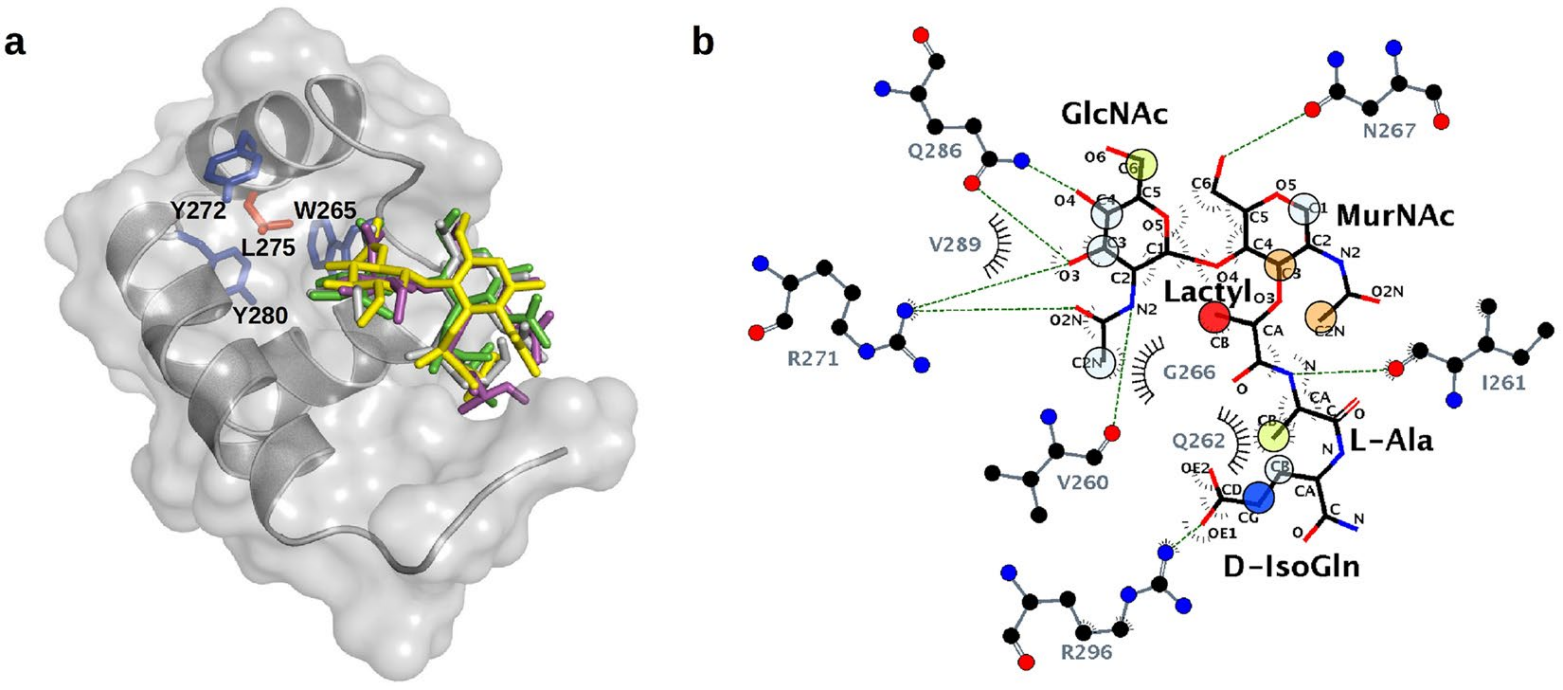
Structural model of the complex of GMDP with the R2 repeat of C-Cpl-7 wt based on docking analysis and STD-NMR data. (**a**) Stick representation of GMDP conformers in the best complex models obtained with AutoDock4.2 (green), CRDOCK (grey), AutoDock Vina (yellow), and DOCK6 (magenta). R2 surface is coloured in grey and protein residues irradiated during STD experiments are drawn as sticks (aromatics in blue and Leu275 in orange). (**b**) Ligplot^80^ representation of the hydrogen bonds and hydrophobic contacts in the R2:GMDP complex generated with AutoDock4.2, the one which agreed best with the GMDP epitope map derived from STD measurements. Overlapping circles show the STD epitope mapping using the colour code of Fig. 6.

Aiming to identify the binding locus of GMDP and the feasible interactions, the structure of the complex was modelled by docking of the ligand onto the crystal structure of R2, using STD data as a guide. A first, “blind docking”^31,32^ carried out with AutoDock4.2, using a grid box that comprised the whole surface of R2, showed that most of the GMDP molecules were docked at a shallow groove placed at the interface of helices α5 and α6, with helix 4 at the rear. None of them was at the opposite face of R2 (Supplementary Fig. S11a). Remarkably, the fully conserved Arg271 substituted by alanine in the crystallized C-Cpl-7R mutant was placed at the centre of this groove. Next, we used four different approaches (AutoDock4.2, CRDOCK, AutoDock Vina and Dock6.3) to dock GMDP onto the R2 structure using a grid box centered on the Nη2 atom of Arg271 side chain (see details in Methods and Supplementary information). The ligand showed very similar poses in the final solutions rendered by the four docking methods (Fig. 7a), with RMSDs of about 0.7 Å for the disaccharide atoms and 2.3 Å for the whole molecule; the more variable part being the D-isoGln residue (Fig. 7a, Supplementary Fig. S11). The *N-*acetyl group of the GlcNAc unit is more deeply docked into the binding site whereas the MurNAc moiety shows a shallower location with its pyranose ring parallel to the R2 surface. Feasible contacts are summarized in Supplementary Table S3. Notably, R2 residues inferred to be involved in GMDP recognition were well conserved in the four final models of the complex (Supplementary Fig. S11). The GlcNAc ring was hydrogen bonded through the hydroxyl groups at positions 3 (with Arg271 and Gln286) and 4 (with Gln286), and by the *N*-acetyl group (with Arg271 and Val260). Besides, the MurNAc unit could mediate hydrogen bonds through the hydroxyl at position 6 (with Asn267) and the carbonyl oxygen of the lactyl group (with Arg296 side chain), and the L-Ala moiety is hydrogen bonded with Ile261 main chain. Additionally, hydrophobic contacts could be formed by the methyls of the lactyl group and the acetamido moiety of GlcNAc (with Val260, Gly266, Val289 or Leu293), or by the methyl group of L-Ala (with Ile261 and Gln262).

**Figure 8:**
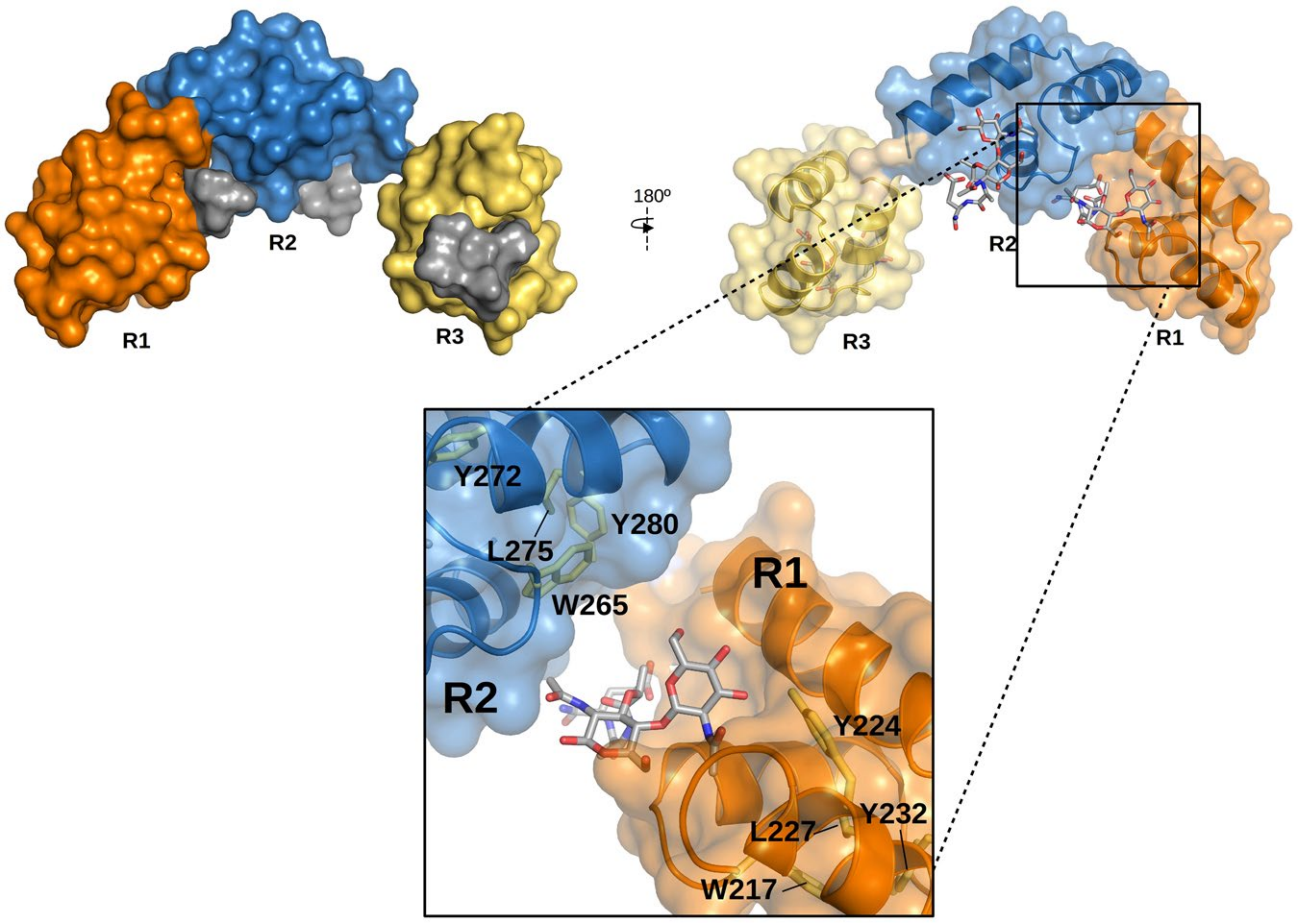
Model of C-Cpl-7 wt in complex with GMDP based on the BUNCH model generated from SAXS data and the three-fold transfer of the GMDP pose in the best docking model to R2 repeat. GMDP molecules are shown in solid surface (left panel) or in stick representation (right panel). A detail of the interface between R1 and R2 repeats is shown in the close-up. R2 likely contributes to shape the binding pocket of R1, thereby explaining the STD signals of the anomeric proton and the acetamido group of MurNAc, unaccounted for in the model of GMDP in complex with one isolated repeat. Protein residues relevant for the STD measurements observable in this view (Y224, Y280, W265 L227, L275) are shown in stick representation.

The model with the fewest violations of the STD-mapping results was provided by AutoDock4.2 (Fig. 7b). The only unaccounted signals were the intense STD peaks due to the anomeric H1 proton (aromatic irradiation) and the acetamido group of the MurNAc unit (Fig. 6d), which were explained by none of the four docking models (Fig. 7; Supplementary Fig. S11). However, when the GMDP molecule was transferred, by superimposition of the GMDP:R2 complex structure, onto every repeat of the BUNCH model, a more complete picture of GMDP recognition by the full-length C-Cpl-7 domain was depicted (Fig. 8). This extended model would be compatible with a 1:3 stoichiometry for the C-Cpl-7/GMDP complex, as the binding-site of each repeat would be accessible to the ligand in the SAXS-derived solution structure. Furthermore, the MurNAc unit bound to either R1 or R2 repeats would be placed in the proximity of the following repeat, with the ring and the *N*-acetyl of MurNAc positioned at a suitable distance and orientation for the transfer of magnetization from the following repeat (R2 and R3, respectively), thereby explaining the previously unaccounted STD signals of MurNAc H1 and acetamide protons. Moreover, the model proposed for the full C-Cpl-7 domain would also explain the different intensity of STD signals obtained by irradiating either the aliphatic region or the aromatic region of the protein, as several aromatic residues of the contiguous repeat would be flanking the other side of GMDP, helping to shape the binding pocket.

**Figure 9:**
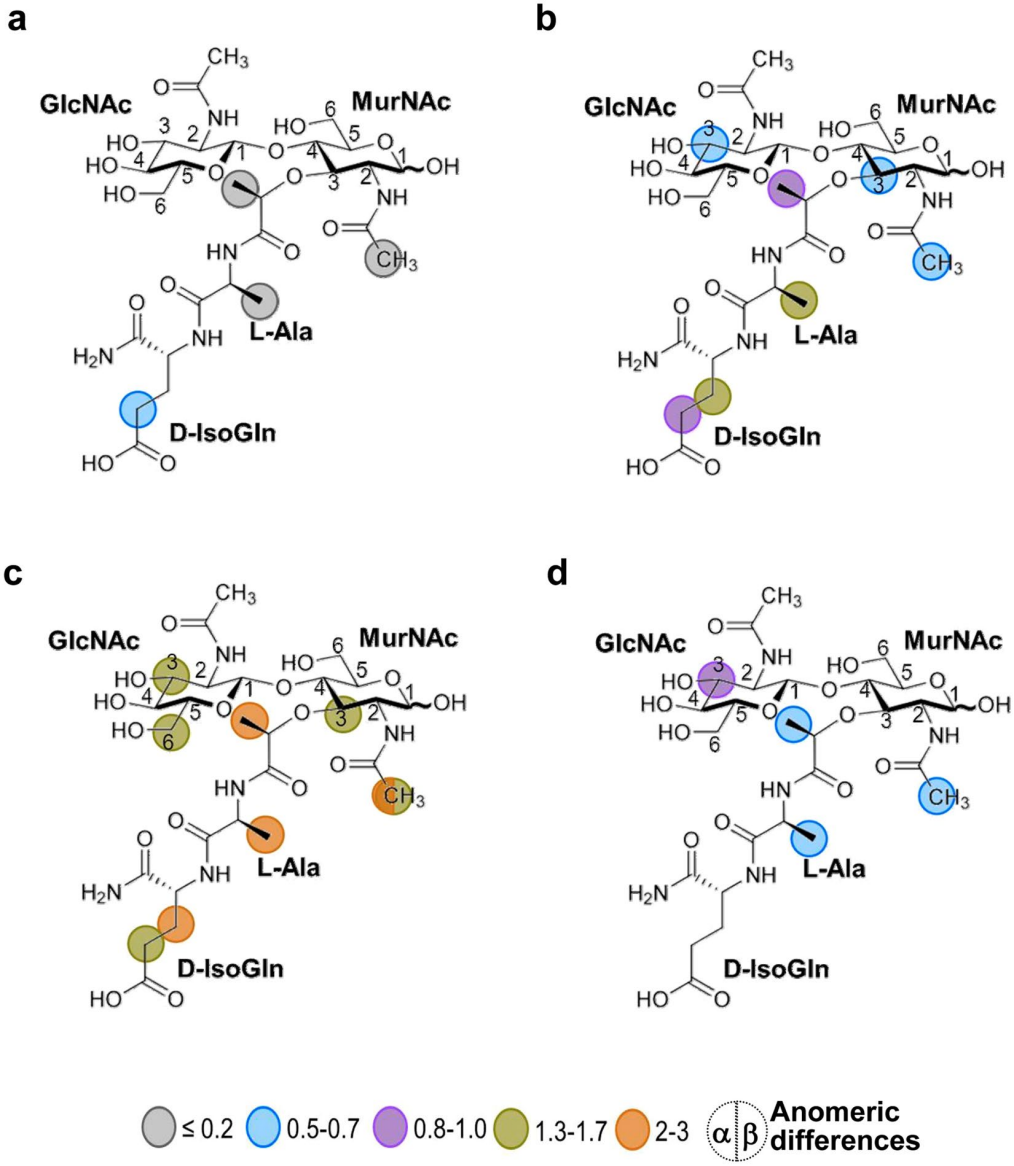
Contribution of the residues mutated in C-Cpl-7R, C-Cpl-7Q and C-Cpl-7I to the GMDP binding. Binding epitope of GMDP in the presence of (**a**) C-Cpl-7R (**b**) C-Cpl-7Q and (**c**) C-Cpl-7I upon irradiation at the methyl region. (**d**) STD-NMR spectrum and binding epitope in the presence of C-Cpl-7I upon irradiation at the aromatic region. The intensity ratio of the STD effect induced by C-Cpl-7R, C-Cpl-7Q and C-Cpl-7I with respect to that of C-Cpl-7 wt is indicated by colours: grey circles **≤**0.2; blue: 0.1–0.5; magenta: 0.8–1.0; green: 1.3–1.7; orange: 2–3. Measurements were carried out in PB-D_2_O buffer at 2.5 mM GMDP and 50 μM mutant concentration.

To asses the docking model of the complex, STD spectra were registered for the three C-Cpl-7 mutants (C-Cpl-7R, C-Cpl-7Q and C-Cpl-7I) in which one residue predicted to be involved in GMDP binding was substituted by alanine in the three repeats (Supplementary Fig. S4). The side chains of the amino acids mutated in C-Cpl-7R (Arg223, Arg217 and Arg319) and C-Cpl-7Q (Gln238, Gln286 and Gln334) were predicted to form hydrogen bonds with the GlcNAc moiety, whereas for C-Cpl-7I (Ile213Ala, Ile261 and Ile309) the hydrophobic contacts with the L-Ala would be altered. Figure 9a–d illustrates the intensity of the STD effects, normalized with respect of those obtained in the presence of C-Cpl-7 wt. The experiments performed in the presence of C-Cpl-7R, used also to solve of the overall domain structure, showed no STD response using aromatic irradiation. In addition, the intensities from the methyl side were extremely weak (~0.05–0.5 times lower than those obtained for the wt protein) and restricted to the methyl protons of L-Ala and the lactyl group, and the γγ’ protons of D-IsoGln (Fig. 9a). By using microarrays binding assays, the effect of C-Cpl-7R mutations on the binding of the domain to pneumococcal cell walls was also assayed, and a decrease of around 50% in the intensity of the binding signals was found in relation to C-Cpl-7 wt (Supplementary Fig. S7e,f). In the presence of C-Cpl-7Q, a notable reduction in the STD signals of the glycosidic protons was found from the methyl side (~0.05–0.7 times lower than for the wt protein), whereas those of the lactil moiety and the peptide stem were unchanged or slightly enhanced (Fig. 9b). Again, no STD response was found using aromatic irradiation. Finally, C-Cpl-7I mutations induced a generalized drop in the STD signals registered upon aromatic side irradiation (~0.05–0.5 times lower than for the C-Cpl-7 wt), except for the proton H3 of GlcNAc that remained practically unchanged (Fig. 9d). In contrast, the intensity of the signals was enhanced when the irradiation was performed from the methyl side (Fig. 9c).

Preservation of C-Cpl-7 wt native structure in C-Cpl-7R has been demonstrated above, and this also seems to be the case of C-Cpl-7Q, according to its CD and ^1^H NMR spectra, hydrodynamic features and thermal stability (Supplementary Figs 7b–d and S9 and Table S4). Therefore, the weakening of the contacts established between the glycanic part of GMDP and the binding surface of C-Cpl-7R or C-Cpl-7Q unveiled by the STD experiments would be coherent with the interactions predicted by the docking models. In contrast, the differences found between the near-UV CD spectra of C-Cpl-7I and C-Cpl-7 wt, covering all the bands of the spectrum, and a down-shift of 11.8 °C in the denaturation temperature of the mutant (Supplementary Fig. S7c,d, and Supplementary Table S4) suggested that substitution of alanine for isoleucine in C-Cpl-7I might have altered the bundle packing. In addition, differences were observed in the the ^1^H NMR spectra of C-Cpl-7I upon GMDP binding, suggesting an induced conformational change. All these facts make difficult the interpretation of STD results of this mutant in terms of binding. Because of the lower size of the alanine side chain and its location at the bottom of the binding locus, a variation in the local conformation of the binding site might have allowed a higher approximation of GMDP to the repeat surface, thereby explaining the increase of the STD signals when using the methyl irradiation.

## Discussion

Nowadays, antibiotic resistance is one of the biggest threats to global health, food security and development. Without urgent actions, we are heading for a post-antibiotic era in which common infections and minor injuries can once again kill. In this scenario, development of new anti-infectives that can be deployed against multi-drug resistant infections is paramount, and recombinant lysins appear as one of the ten best alternatives to conventional antibiotics^9^. Actually, the first commercial lysin (Staphefekt) is already on the market for topical treatment of skin infection by methicillin-resistant *Staphylococcus aureus* (MRSA) (http://www.micreos.com/). Despite the primary role of cell wall recognition domains in lysin selectivity as antibacterials, limited information is available about respective surface receptors or the impact of domain sequence variability. Their understanding would lead to improved versions of lysins for therapeutic application and infection prevention. Here, we have solved the structure of the CW_7 repeats of the Cpl-7 endolysin and demonstrate that they recognize and bind GMDP, a fragment of the PG building block, as previously suggested^28^. Also, a structural model of the domain in complex with GMDP consistent with the STD-mapping of the ligand epitope and mutational studies has been proposed, and sequence variability within CW_7 family analyzed.

This broadly distributed cell wall-binding motif folds into a three-helix bundle stabilized by hydrophobic and polar interactions mediated by residues highly conserved among sequences of the CW_7 family, and by two conserved aromatic residues capping the bundle side defined by the two first helices (see Supplementary Fig. S2). Crystals of the C-Cpl-7R mutant showed a tight packing of the three CW_7 repeats in the overall structure and a minor impact of the arginine-to-alanine mutation on the bundle structure. Interestingly, SAXS characterization of the domain structure in solution revealed a more open conformation (solution values of *D*_max_ and *R*_g_ were ∼1.37-fold higher than those calculated from the crystallographic coordinates), which was compatible with the hydrodynamic behaviour of C-Cpl-7 wt and C-Cpl-7R in sedimentation experiments and the low complexity of their 1D ^1^H NMR spectra. Taking into account the high overall net charge of the domain (−14.27 at neutral pH), it seems plausible that electrostatic repulsions among the repeats could favour the extended structure found at the low ionic strength (*I* = 0.044) of PB buffer. In line with this, the strong shielding of charges provided by the high ionic strength (*I* ∼ 6.6) of the crystallization buffer may have facilitated the structural compaction found in the full-length C-Cpl-7 crystals.

By STD-mapping of the GMDP binding epitope we have proved that the two saccharide units and the dipeptide are recognized by the binding site. This observation agrees with the previous finding that neither GlcNAc oligosaccharides nor MurNAc-L-Ala-D-isoGln stabilized C-Cpl-7 against thermal denaturation^28^. Moreover, it further suggests that GMDP might be the minimal element of the PG recognized by the CW_7 repeats. Interestingly, our docking studies pointed out that each CW_7 repeat may contain a muropeptide binding site in a shallow groove located between the two last helices of the bundle and the first one at the bottom. The favourable contacts inferred from the R2:GMDP docking model largely agreed with the mapping of the ligand epitope, as already noted, and the repeat residues involved in GMDP binding were all well conserved in the CW_7 family (Supplementary Fig. S2). Moreover, the strong drop of STD signals in the presence of C-Cpl-7R or C-Cpl-7Q supports the implication of Arg271 and Glu286, and the equivalent residues of R1 and R2, in the complex stabilization (see Supplementary Table S3) and validated as well the binding locus. On the other hand, the nature of the feasible interactions depicted by the docking model suggests that the drop in GMDP binding detected by STD-NMR at high ionic strength might entail a reduction in the binding site accessibility, as it occurs in the crystal structure of the full-length domain. In this context, it is tempting to speculate whether the ability of adopting either a close or an open conformation might constitute a mechanism for modulating Cpl-7 lytic activity, alike to those reported for other endolysins^33–35^. In line with this, Cpl-7 activity on pneumococcal cell walls decreased drastically as the solution ionic strength was increased^36^, and GMDP binding was impaired as well, as determined by STD-NMR.

Remarkably, the extension of the GMDP binding mode predicted for a single repeat to the BUNCH model of the full-length domain indicated that the solution structure was compatible with a 1:3 C-Cpl-7-to-GMDP molar ratio stoichiometry, as the three potential binding sites of the domain were fully accessible for GMDP binding. Moreover, according to this model, the inter-repeat linker and the N-terminal proximal region of the following repeat could help to shape the GMDP binding sites in multimodular CW_7 domains. Such disposition would also account for *i*) the inclusion of Arg296 (from the R2-to-R3 linker) among the residues in contact with GMDP by docking studies, *ii*) the complete STD-map of the GMDP binding epitope, and *iii*) the differences found in the STD signals upon irradiation of either the methyl region or the aromatic region of the C-Cpl-7 domain. Thus, our results support the notion that every CW_7 repeat of C-Cpl-7 provides a binding site for the bacterial PG rather than acting together as a whole to generate a tertiary structure essential for PG recognition. This hypothesis is further supported by the ability of the Cpl-7Δ2 mutant, comprising only one CW_7 repeat, to hydrolyze pneumococcal cell walls 780-fold faster than the separate catalytic model, and with the strong increase in activity accompanying the acquisition of another repeat in Cpl-7Δ1 (Fig. 1). Interestingly, the superimposition of the NMR-based structure of the PG network^37^ onto the C-Cpl-7:(GMDP)_3_ model showed that the three-fold symmetry of the PG backbone and the spatial arrangement of the stem peptides would likely allow the simultaneous attachment of three PG chains to the C-Cpl-7 domain (Supplementary Fig. S12). The possibility that contacts between the C-Cpl-7 domain and the PG network could extent to other muropeptide units or to additional residues of the peptide stem or the inter-peptide bridges cannot be presently dismissed.

Targeting of the PG by CW_7 motifs accounts for Cpl-7 ability to kill both Gram-positive and Gram-negative bacteria^17^. However, Cpl-7 does not behave as a broad-range killing lysin, and there is not an evident relationship between the type of PG structure and Cpl-7 bacteriolytic activity. PGs of susceptible bacteria so far identified are of type A3α with L-(Ala/Ser)-L-Ala or L-Ala_2/3_ linking-peptides (*e*.*g*. *S*. *pneumoniae*, *E*. *faecalis* or *S*. *pyogenes*), A1α (*Streptococcus suis*), or A1γ (*E*. *coli* and *Pseudomonas putida*). However, neither *Listeria monocytogenes* (A1γ PG type)^38^ nor *Streptococcus agalactiae* (A3α PG with L-Ala-L-(Ala/Ser) linkers) or *Streptococcus mutans* (A3α PG with L-Thr-L-Ala bridges)^39^ are killed by Cpl-7^17^. Thus, Cpl-7 specificity is likely modulated by secondary factors that help to define charge distribution and PG accessibility, like capsular components, PG local composition, or surface attached molecules^17,40^.

Tailoring of sequences and domain composition at genetic level has been required to achieve the high substrate selectivity that constitutes one of lysins’s strengths as anti-infective drugs. The inspection of CW_7 sequences of protein databases has revealed a well conserved three-residue deletion in sequences from eubacteria in comparison with those of Firmicutes (Supplementary Figs S2 and S3). Deviations from this trend are localized in a few architectures (Supplementary Figs S2 and S3). In addition, an uneven distribution of major CW_7-containing architectures among bacterial phyla and/or genus has become evident (Supplementary Fig. S1). Moreover, we have found that fine traits of sequence conservation among repeats can vary with the type of architecture, as also occurs with the linkers connecting the CW_7 repeats, whose length can depend too on the phyla and genus (Supplementary Fig. S3). Taken together, all these data point to a co-evolution of the CW_7 sequences with those of catalytic domain/s and, when present, additional cell wall-binding domains. By means of it, small differences in the affinity of a separate domain, or domains, may have been amplified –via multiple domain/cell wall interactions– to alter the efficiency and substrate specificity, even at the species or strain level. Of note, the acquisition of one LysM module by GH25-(CW_7) sequences changed its predominance on Firmicutes towards Actinobacteria, whereas the GH25-(CW_7)-LysM_2_ type is so far confined to Bifidobacteria (Supplementary Fig. S1). It has been proposed that LysM modules might have evolved to ensure optimal recognition of chitine and PG glycosidic chains, with peptide stems acting as negative modulators of LysM binding to PG^41^. Hence, specific combination of CW_7 and LysM modules in a single polypeptide chain might have facilitated a rapid evolution to new specificities while using existing cell-wall surface receptors. The same might apply to the great variety of architectures shown by the CW_7 containing proteins. Moreover, domains and linkers co-adaptation may be a key issue in their exploitation –either wild-type or in chimeric constructions– to control pathogenic bacteria and to prevent the killing of normal microbiota.

## Methods

### Cloning, expression and purification of proteins

The Cpl-7 endolysin was produced and purified as previously described^11^. The synthetic DNA fragments encoding Cpl-7Δ1, Cpl-7Δ2, C-Cpl-7 wt without tag and the triple (Arg223Ala, Arg271Ala, Arg319Ala) C-Cpl-7R, (Gln238Ala, Gln286Ala and Gln334Ala) C-Cpl-7Q, and (Ile213Ala, Ile261Ala and Ile309Ala) C-Cpl-7I mutants were purchased from ATG:biosynthetics (Merzhausen, Germany) as *E*. *coli* codon-optimized, pUC-derivative recombinant plasmids. The amino acid sequences of respective proteins are shown in Supplementary Fig. S4. The relevant DNA fragments were subcloned into the pT7-7 expression vector^42^ using NdeI and EcoRI (Cpl-7Δ1 and Cpl-7Δ2) or NdeI and PstI (C-Cpl-7 wt and C-Cpl-7R, C-Cpl-7Q and C-Cpl-7I), giving rise to plasmids pT7Δ1, pT7Δ2, pTCCpl7, pTCCpl7R, pTCCpl7Q and pTCCpl7I, respectively. For protein overproduction, *E*. *coli* BL21(DE3) cells transformed with these plasmids were incubated in LB medium containing ampicillin (0.1 mg/ml) to an optical density at 600 nm (OD_600_) of about 0.6. Then, IPTG (100 μM) was added and incubation proceeded overnight at 30 °C, except for the C-Cpl-7 mutants that were induced at 20 °C. Respective proteins were purified using the protocol reported for the full length protein^11^ with small variations in the salt gradients applied to elute the different proteins. In parallel, the C-Cpl-7 coding gene was amplified by PCR from the pCP700 plasmid containing the Cpl-7 gene^43^ using NC-Cpl-7 forward (5′-G**GAATTC**AACAATGAAAACACTCTAAAAAGCCTTACC-3′) and CC-Cpl-7 reverse (5′-CCC**AAGCTT**AAAATAGCTAGTAGAAAATTTCTACTAGCTTTTACTTGTTA-3′) primers, where nucleotides in bold are restriction sites for EcoRI and HindIII, respectively. The 500 bp PCR product was cloned into the pKLSL_t_ vector^44^ and the resulting plasmid pKLSLt-C-Cpl-7 was transformed into *E*. *coli* BL21 (DE3) cells. Cells expressing the LSLt-C-Cpl-7wt fusion protein were grown at 37 °C in LB medium containing kanamycin (50 μg/ml) to an OD_600 = _0.8 and induced with IPTG (50 µM) for 12 h at 16 °C. Purification of the LSLt-C-Cpl-7 fusion protein was carried out by affinity chromatography onto a Sepharose 4B affinity column (GE Healthcare) followed by LSLt fusion-tag cleavage with the TEV protease^45^. The accuracy of all cloned genes was confirmed by DNA sequencing (Secugen S.L., Madrid). All purification steps were performed at 4 °C and the purity and masses of isolated proteins were checked by SDS-PAGE (12–15% acrylamide/bis-acrylamide) and MALDI-TOF (matrix-assisted laser desorption/ionization-time of flight) before storage at −20 °C. Unless otherwise stated, proteins were dialyzed at 4 °C against the appropriate buffer and centrifuged for 5 min at 11,600 × *g*. Theoretical absorption coefficients at 280 nm (ProtParam; web.expasy.org/protparam/) were used to determine protein concentrations.

### Activity assays

Cell wall solubilisation assays were carried out at 37 °C in PB buffer (pH 6.0) using [*methyl*-^3^H]choline-labeled pneumococcal cell walls from strain R6 as substrate^46^ as described elsewhere^17^. One unit of activity was defined as the amount of enzyme that catalyses the solubilization of 1 µg of cell wall material in 10 min. Data are the average of three independent assays with four to six replicas each.

### Mass spectrometry

MALDI-TOF measurements were performed on a Voyager DE-Pro mass spectrometer (Applied Biosystems) as described^28,47^. Cpl-7, Cpl-7Δ1 and Cpl-7Δ2 spectra were obtained over the *m/z* range of 16–50 ku, employing carbonic anhydrase and enolase from *Saccharomyces cerevisiae* (Sigma) for external mass calibration. C-Cpl-7 wt and mutant spectra were obtained over the *m*/*z* range 2–20 ku. Insulin (bovine), thioredoxin (*E*. *coli*, oxidized) and apomyoglobin (horse skeletal muscle) (Calibration Mixture 3 of Sequazyme Peptide Mass Standards Kit; Applied Biosystems) were used in this case for external mass calibration.

### Analytical ultracentrifugation

Sedimentation velocity experiments were carried out at 20 °C in an Optima XL-A analytical ultracentrifuge (Beckman Coulter). Measurements were performed in PB buffer at 45,000 rpm using cells with double sector Epon-charcoal centrepieces (AN50Ti rotor). Differential sedimentation coefficients were calculated by least-squares boundary modelling of the experimental data, and corrected to *s*_20,w_ values with the program SEDFIT^48^. The ratio of the frictional translational coefficients of the protein particle and the equivalent rigid sphere (*f*/*f*_0_) and the Stokes’ radii (*R*_s_), related to the protein hydrodynamic shape, were calculated with the SEDNTERP program^49^, using as input data the *s*_20,w_ value, together with the partial specific volume and the hydration coefficient calculated from the amino acid sequence using SEDNTERP.

### Circular dichroism

CD measurements were performed in PB buffer at 20 °C in a J-810 spectropolarimeter (Jasco Corporation) equipped with a Peltier temperature control system, using protein concentrations of 0.17 mg/ml (far-UV) and 0.56 mg/ml (near-UV). Spectra were recorded and analysed as described^50^. Thermal denaturation experiments were carried out by increasing the temperature from 20 to 90 °C at a scanning rate of 60 °C/h and ellipticity variations at 222 nm were monitored every 0.2 °C. The best fitting parameters of the two-state transition model^28^ to the denaturation profiles are shown in Supplementary Table S4.

### Crystallization and X-ray structure determination

The strategy to obtain suitable crystals of C-Cpl-7 and the structure determination is described in Supplementary Methods. Briefly, crystals containing the R_1–2_ fragment (see Fig. 1) grew at 18 °C using the hanging-drop vapour-diffusion method by mixing 2 µl of protein solution (20 mg ml^−1^ of C-Cpl-7 wt purified from the LSLt-C-Cpl-7 fusion protein in 25 mM Tris, pH 8, and 100 mM NaCl) with 2.5 µl of reservoir solution (1.3 M trisodium citrate) and including 0.5 µl 1 M HEGA-8 (octanoyl-*N*-hydroxyethylglucamide) as additive, equilibrating against 500 µl of crystallization solution in the reservoir. Crystals of the full length C-Cpl-7R triple mutant were obtained by mixing 2 µl of protein solution (20 mg ml^−1^ in the buffer used for the wild type protein) with 2.5 µl reservoir solution supplemented with 0.5 µl 1 M HEGA-8, equilibrating against 500 µl of crystallization solution (1.1 M trisodium citrate) in the reservoir. Both protein crystals were soaked for a few seconds in a cryoprotectant solution prior to flash-cooling at 100 K in liquid nitrogen. X-ray data sets were collected on beamline ID 14-1 (λ = 0.934 Å) at the ESRF (Grenoble, France) and on Bl-13-XALOC (λ = 1.05739 Å)^51^ at ALBA synchrotron (Barcelona, Spain). Data were collected to 1.67 Å resolution from the R_1-2_ crystal, and up to 2.8 Å resolution for the full-length C-Cpl-7R crystal. Data sets were processed using *XDS* ^52^ and iMOSFLM, and scaled with AIMLESS from CCP4 program suite^53^. The structure of a single repeat from R_1-2_ crystals (referred as R2) was solved with *ARCIMBOLDO* ^22^ that combines fragment location with PHASER^54^ and extension through density modification and autotracing with SHELXE^55^. C-Cpl-7R structure was determined by molecular replacement using the programme *MOLREP* ^56^ and the structure of the repeat from R_1-2_ crystals as initial model. Manual model building was performed using COOT^57^. Models were refined using PHENIX suite^58^. Excellent density maps were obtained for the single repeat structure (Supplementary Fig. S6) whose refinement converged to the final values of R = 0.17 and R_free = _0.22. Full-length C-Cpl-7R structure also presented an excellent electron density map along the sequence except for the last residue of the polypeptide chain. Refinement of C-Cpl-7R resulted in an R factor of 0.20 and R_free_ of 0.29. The final models presented a good stereochemistry with 100% (R2) and 98% (C-Cpl-7R) of the non-glycine, non-proline residues found in the most favoured regions, and with 0% (R2) and 0.71% (C-Cpl-7R) residues in disallowed regions. Data processing results and refinement statistics are summarized in Table 2. PDB’s accession codes for R2 and C-Cpl-7R are 4CVD and 5I8L, respectively.

**Table 2:** X-ray data collection and refinement statistics.

**Diffraction data statistics** ^**a**^		
	CW_7 wt repeat (R2)	C-Cpl-7m
*Wavelength* (Å)	0.933400	1.05739
*Space group*	P6_1_	P2_1_2_1_2_1_
*a*, *b*, *c* (Å)	50.40, 50.40, 28.54	29.52, 50.33, 86.10
α, β, γ	90, 90, 120	90, 90, 90
*Resolution range* (Å)	28.54–1.67	43.04–2.8
	(1.76–1.67)	(2.82–2.8)
*Unique reflections*	4927 (701)	3562 (475)
*Completeness* (%)	99.68 (98.74)	83.4 (93.7)
*Redundancy*	10.70 (10.40)	3.8 (3.7)
*R*_*merge*_	0.05 (0.18)	0.05 (0.23)
*R*_*pim*_	0.012 (0.06)	0.03 (0.14)
Average I/σ(I)	30.96 (13.10)	9.5 (4.8)
**Refinement statistics**		
*Resolution range* (Å)	28.54–1.67	15.00–2.8
R_work_/R_free_	0.17/0.22	0.20/0.29
***N*** ^***o***^ ***Atoms***		
Protein	370	1085
Water	32	8
Ligand	—	6
***B-factor*** (**Å** ^**2**^)		
Protein	10.50	22.10
Water	17.00	15.10
Ligand	—	22.40
***R***.***m***.***s***. ***deviations***		
Bond length (Å)	0.008	0.007
Bond angles (°)	1.20	1.42
Ramachandran favoured/outliers (%)	100/0	98.0/0.71
Residues in the AU	48	143
PDB code	4CVD	5I8L

^a^Values in parenthesis are for the highest resolution shell.

### NMR spectroscopy

NMR spectra were acquired at 25 °C on a Bruker Avance 800 MHz spectrometer equipped with inverse triple-resonance TCI cryo-probe and pulse gradients, using samples prepared in 500 μl of 99.9% D_2_O-buffers. ^1^H NMR spectra of all C-Cpl-7 constructs were very well dispersed and shared a very up-field methyl signal (−1 ppm) with full-length Cpl-7 (Supplementary Fig. S9), which confirmed the retention of the native fold. STD-NMR experiments were optimized as described^59^ using pulse sequences reported in the literature^60^. Briefly, experiments were carried out using a train of 50 ms Gaussian shaped pulses of flipping angles of 650° (height 87.7 Hz) and 2.0 s of total saturation time. The on-resonance frequency was set around −1.0 ppm (aliphatic region) or 7.7 ppm (aromatic region) whereas the off-resonance frequency was –150 ppm, where no protein signals are present. Based on chemical shift calculations with programs SHIFTX^61^ and SIFTSS 4.0^62^ using the coordinates of R2 structure, the upfield methyl signal at about −1 ppm was proposed to correspond to Leu227 (R1), Leu275 (R2) and Leu323 (R3), in accordance with their burial in the hydrophobic core in proximity to aromatic groups, whereas the 7.7 ppm aromatic signal would arise from protons of Trp217, Tyr224, and Tyr232 in R1, Trp265, Tyr272, and Tyr280 in R2, and Trp313, Tyr320, and Tyr328 in R3. Blank experiments were performed to assure the absence of direct saturation to the ligand protons. Samples for STD were prepared at protein/GMDP ratios of *ca* 1:50 (0.033–0.05 mM protein range) in PB-D_2_O buffer (pD 7.1), or in 0.25 M sodium pyrophosphate, 1.5 M NaCl (pD 8.6). The relatively low complexity of C-Cpl-7 wt and mutant spectra in PB suggested that, at low salt concentration, the repeats behave as independent units in which protons occupying equivalent positions have very similar chemical environments.

To map the binding epitope of GMDP (Merck), STD intensities were normalized with respect to the one of the highest response, taken as 100%. The complete assignment of the ^1^H NMR spectra of the *α*- and *β*-anomers of GMDP in D_2_O, required for epitope mapping, was done on the bases of 2D spectra COSY, TOCSY (50 ms mixing time) and ROESY (125 ms mixing time), following standard procedures. STD-TOCSY experiments were acquired using 256 increments and an isotropic mixing time of 60 ms.

### Docking studies

The coordinates of R2 structure (PDB: 4CVD) were used for the receptor. The hydrogen atoms and partial charges at pH 7.0 were added with the pdb2pqr software^63^. The coordinates of the ligand, GMDP, were obtained from its complex with the *S*. *pneumoniae* autolysin LytC (PDB: 2WWD). The GLYCAM06 (Glycam_06 g.dat downloaded from http://glycam.org/ForceField.jsp)^64^ and PARM99SB^65^ parameters were used to optimize the ligand and receptor geometries, respectively. The MurNAc unit, undefined in GLYCAM06 force field, was treated as a GlcNAc unit with a lactyl group bound at position 3, which was optimized *ab initio* using Gaussian 03^66^ at the HF/6–31 G level of theory. First, a “blind docking”^31,32^ of GMDP into the crystal structure was run with AutoDock4.2 (100 runs with default parameters), using a grid box that comprised the whole surface of R2, in order to find the most probable binding cavity. Next, and to cover the widest range of possible solutions compatible with STD data, docking programs that use different docking and scoring algorithms were employed: CRDOCK^67^, DOCK6.3^68^, AutoDock4.2^69^ and AutoDock Vina^70^ (see Supplementary information). After default evaluation by the respective methods, the best scored solutions were carefully analyzed using the STD data as a quality control of the ligand pose in the complex. The binding model with lowest docking energy and highest compatibility with STD data from each method was selected and minimized *in vacuo* by 2500 cycles of conjugate gradient followed by 500 cycle of steepest descent, using the Sander program (AMBER12 package)^65^. Finally, the four minimized solutions were compared and the one that best accounted for the STD-mapping results was proposed as the most probable model of the GMDP:R2 complex.

### Small-Angle X-Ray Scattering

SAXS measurements were performed at 20 °C at the BL11-NCD beamline at ALBA synchrotron (Barcelona, Spain). Before data acquisition, the protein was extensively dialyzed against PB buffer, centrifuged (20 min × 50,000 rpm) at 4 °C in a Beckman TL-100 (TL100.3 rotor), and the lowest part of the sample was discarded to remove possible aggregates. Protein spectra were acquired at 7.6, 3.8 and 1.9 mg/ml. The wavelength used was 0.995 Å, and the sample to detector distance (Quantum 210r; ADSC, California) 2.0 m, which allowed collecting data from 0.01 to 0.55 Å^−1^ of the scattering vector, *q*, defined as 4π·sinθ/λ, where θ is the scattering angle. No changes by radiation damage were detected in the 30 frames of 50 ms each recorded per sample, which were averaged and normalized by beam intensity and detector response. Buffer scattering was subtracted from the protein data. Corrected spectra were then normalized and extrapolated to infinite dilution, as they slightly bended down at very low values of *q* with increasing protein concentrations (Supplementary Fig. S8a), which is indicative of some inter-particle repulsion. Data were then processed and analyzed using the ATSAS package^71^. *R*_g_ was calculated by the Guinier approximation using PRIMUS and the pair-of distances-distribution function (*P*(*r*)) implemented in GNOM^72^. *P*(*r*) yielded also *D*_*max*_ value. Ten *ab initio* bead models of C-Cpl-7 were constructed with DAMMIF^25^, superimposed with SUPCOMB, filtered by spatial discrepancy with DAMFILT (the spatial discrepancy parameter was 0.57 ± 0.07 Å), and averaged with DAMAVER^73^. Rigid body modelling of the solution structure was performed with the BUNCH program^26^ using as input the coordinates of the repeats in C-Cpl-7m crystal structure. The repeats were moved and rotated as independent rigid bodies, whereas the last four residues of inter-repeat linkers (residues 249 to 252 and residues 297 to 300) were treated as a chain of dummy residues. Distances between the residues forming the two inter-domain salt bridges (Arg248/Glu259 and Arg296/Glu307) found in the crystal structure were included as structural restraints. The EOM method^27^ was used to analyze the flexibility of the C-Cpl-7 domain in solution. The repeat coordinates, linker definition and restraints employed in BUNCH were maintained. The theoretical scattering profiles of the SAXS-derived models and the crystal structure of the C-Cpl-7R mutant were calculated with CRYSOL^24^, and the theoretical hydrodynamic parameters with WinHydropro^74^.

### Preparation of cell wall microarrays

For preparation of robotic arrays, cell wall samples from *S*. *pneumoniae* R6, with choline- or ethanolamine-containing (lipo)teichoic acids, and *Micrococcus luteus* (Sigma) were printed on 16-pad nitrocellulose-coated glass slides (FAST-slides, Maine manufacturing) using a non-contact arrayer (Sprint, Arrayjet Ltd.). Pneumococcal cell walls were prepared, as described^75^, from cultures grown without shaking at 37 °C to an OD_620_ of 0.5 in C medium supplemented with yeast extract (0.8 mg/ml; Difco Laboratories) or in ethanolamine-containing Cden-EA medium^76,77^. Cell wall sample suspensions in PBS were diluted with two volumes of 70.5% glycerol, 0.09% Triton X-100 (final concentrations 47% and 0.06%, respectively) and printed in a four-level dose-response format by applying 100 pl/spot. Spots were printed as triplicates. 1 µl of Cy3 fluorophore (GE Healthcare) was added per millilitre of sample suspensions, to enable post-array monitoring of the spots^78^ by scanning fluorescence signals upon excitation at 532 nm (green laser) with a GenePix 200-AL scanner (Axon, Molecular Devices).

### Microarray binding assays

C-Cpl-7 wt and C-Cpl-7R were biotinylated using the ECL protein biotinylation module (Amersham). Briefly, 200 μl of protein (1 mg/ml) were incubated with the biotinylation reagent according to manufacturer’s protocol, except that reagent removal after labelling was performed by exhaustive dialysis. The printed slides were blocked for 1 h with 0.25% (v/v) Tween-20 in PB buffer, pH 6.0, at 20 °C. Then, microarrays were rinsed with PB and overlaid with a solution of 0.6 μM biotinylated C-Cpl-7 wt or C-Cpl-7m in overlay buffer (0.1% (v/v) Tween-20 in PB buffer, pH 6). After incubation for 1.5 h at 20 °C, microarrays were washed 4 times with PB and binding was detected by incubating with AlexaFluor-647 (AF647)-labelled streptavidin (Invitrogen) at 1 μg/ml in overlay buffer for 35 min at 20 °C. The slides were washed thoroughly with PB and subsequently with water and scanned for AF647 fluorescence signals (excitation at 635 nm, red laser).

### Data availability

The datasets generated during and/or analysed during the current study are available from the corresponding author on reasonable request.

## Electronic supplementary material

Supplementary Information
