# Supplementary material for: Deciphering how Cpl-7 cell wall-binding repeats recognize the bacterial peptidoglycan
Source: Sci Rep. 2017 Nov 28;7:16494. doi: 10.1038/s41598-017-16392-4 (PMC5705596; doi:10.1038/s41598-017-16392-4)
Supplement: Supplementary file 1 — Supplementary Information [file 41598_2017_16392_MOESM1_ESM.pdf]

Supplementary Information

Deciphering how Cpl-7 cell wall-binding repeats recognize the bacterial peptidoglycan

Noemí Bustamante[1,2‡], Manuel Iglesias-Bexiga[1,2‡], Noelia Bernardo-Garcia[1], Noella Silva-Martín[1], Guadalupe García[1,2], María A. Campanero-Rhodes[1,2], Esther García[2,3],Isabel Usón[4], Rubén M. Buey[5], Pedro García[2,3], Juan A. Hermoso[1], Marta Bruix[1], Margarita Menéndez[1,2*]

[1]Instituto de Química-Física Rocasolano, Consejo Superior de Investigaciones Científicas, Serrano 119, 28006, Madrid, Spain.

[2]CIBER de Enfermedades Respiratorias (CIBERES).

[3]Centro de Investigaciones Biológicas, Consejo Superior de Investigaciones Científicas, Ramiro de Maeztu 9, 28040, Madrid, Spain.

[4]Instituto de Biología Molecular de Barcelona, CSIC, Baldiri Reixach 13, 08028 Barcelona, Spain, and ICREA (Institució Catalana de Recerca i Estudis Avançats)

[5]Metabolic Engineering Group. Departamento de Microbiología y Genética, Universidad de Salamanca, Campus Miguel de Unamuno, 37007, Salamanca, Spain.

*Correspondence to: Margarita Menéndez (mmenendez@iqfr.csic.es).

‡These authors contributed equally to this work.

Supplementary methods

Crystallization

Crystallization was performed using a high-throughput NanoDrop™ ExtY robot (Innovadyne Technologies Inc., California, USA). The initial crystallization screening kits were JCSG+ Suite, PACT Suite (Qiagen, Düsseldorf, Germany), Index HT and Crystal Screen (Hampton Research, Aliso Viejo, California, USA). All crystallization nano-trials were performed by the sitting-drop vapour-diffusion method at 291K using Innovaplate™ SD-2 microplates (Innovadyne Technologies Inc.). Crystallization was performed mixing equal amounts (250 nl) of protein (20 mg ml^{-1} in 25 mM Tris, 100 mM NaCl, pH 8) and precipitant solutions. NanoDrop robot drops (500 nl, protein and precipitant solutions) were equilibrated against 65 µl of mother liquor.

Small crystals grew from C-Cpl-7 wt protein isolated from LSLt–C-Cpl-7 construction in condition B11 from JCSG Suite, consisting in a reservoir solution of 1.3 M tri-sodium citrate. Positive results were scaled up using the hanging-drop vapour-diffusion method in Linbro plates (Hampton Research). Drops were prepared by mixing 2 µl protein solution with 2.5 µl reservoir solution, including 0.5 µl 1M HEGA-8 (Octanoyl-N-hydroxyethylglucamide) from Detergent Screen (Hampton Research) as additive, equilibrating against 500 µl crystallization solution in the reservoir. Optimized crystals grew in 1.3 M tri-sodium citrate.

Crystals from the full-length protein C-Cpl-7R, in which arginines 223, 271 and 319 are mutated to alanine, were obtained by mixing 2 µl protein solution with 2.5 µl reservoir solution supplemented with 0.5 µl 1M HEGA-8 as an additive, equilibrating against 500 µl crystallization solution in the reservoir. Crystals grew in 1.1 M tri-sodium citrate.

X-ray data collection and structural determination. Both types of crystals were soaked for a few seconds in a cryoprotectant solution (20% v/v glycerol diluted in crystallization mother liquor) prior to flash-cooling at 100 K in liquid nitrogen. X-ray data sets were collected using an ADSC (Area Detector Systems Corporation) Quantum Q315r detector on beamline ID 14-1 (λ = 0.934 Å) at the ESRF (Grenoble, France) for C-Cpl-7 wt ($R_{1-2;}$ see below) crystals, and a PILATUS 6M detector on XALOC beamline Bl-13-XALOC (λ = 1.05739 Å)[1] at ALBA synchrotron (Barcelona, Spain) for those of C-Cpl-7R.

An X-ray data set was collected to 1.67 Å resolution from a C-Cpl-7 wt crystal. It belonged to the hexagonal space group $P6_1$, with unit-cell parameters a = 50.40, b = 50.40, c = 28.55 Å. Despite the fact that the complete C-Cpl-7 wt domain was isolated from the fusion protein LSLt–C-Cpl-7wt, the size and symmetry of the unitary cell were incompatible with the presence of the three repeats in the crystallised fragment. The analysis of its N-terminal sequence (GEFNNENT) and molecular mass (12,156 Da) showed that it was made up of the R1 and R2 repeats (R_{1-2} fragment; see Supplementary Fig.S4), likely due to the hydrolysis of the R3 repeat during crystallisation. Besides, considering the cell volume, the Matthews coefficient calculated for a single repeat and the inter-repeat connecting linker (2.34 Å Da^{-1}), and for a solvent content of 47.40 %, the spatial group ($P6_1$) would admit only one of the two identical repeats (referred to as R2) within the cell-unit. Crystals from the full-length C-Cpl-7R yielded a X-ray data set that diffracted up to 2.8 Å resolution. The crystal belonged to the orthorhombic space group $P2_12_12_1$, with unit-

86.10 Å. Data sets were processed using *XDS* (Kabsch 1988) and iMOSFLM and scaled using AIMLESS from CCP4 program suite[2].

R2 structure was solved with *ARCIMBOLDO*[3] on a large number of alternative fragments derived from random trimming of PDB entries containing partial structures with the highest sequence similarity. The successful attempt was part of a pool of trials with polyalanine fragments made up of 114 residues cut from the entry**.** C-Cpl-7R structure was determined by using R2 structure as initial model in the molecular replacement method using *MOLREP*[4].

Manual model building was performed using COOT[5]. Models were refined using PHENIX suite[6]. Excellent density maps were obtained for the single repeat R2 structure (Supplementary Fig. S6), and refinement converged to the final values of R = 0.17 and R_{free} = 0.22 for the R2 structure. The full-length C-Cpl-7R structure also presented an excellent electron density map along the sequence, except for the last residue of the polypeptide chain. One glycerol molecule, used as cryoprotectant, was identified in the electron density map involved in crystal contacts. Refinement of C-Cpl-7R resulted in an R factor of 0.20 and R_{free} of 0.29.

Docking studies. Flexible docking of the GMDP muropeptide to R2 wild-type structure was carried out using four different methods (CRDOCK, DOCK6.3, AutoDock4.2 and AutoDock-Vina) with distinct docking and scoring algorithms. CRDOCK applies the matching triangle docking method[7]. The conformational sampling of the ligand was performed by clustering, after 100 ps equilibration and 20 ns molecular dynamic simulation of the molecule immersed into explicit solvent using the sander module of AMBER12 suite[8]. The receptor was treated as a rigid molecule, and a three-dimensional grid (50 × 50 × 50 points) with 0.5 Å of grid spacing was centered on the position occupied by the Nη2 atom of the Arg271 side chain. Sigmoidal distance-dependent dielectric constants and original Lennard–Jonnes and hydrogen-bonding potentials were employed to create the grid maps used to dock every GMDP conformer in the receptor. The 50 lowest energy poses were ranked according to a combination of CRDOCKscore, ChemScore and RankScore implemented by the CRDOCK program. The DOCK6.3 method uses the anchor and grow methodology. The ligand and the grid maps were prepared by using automated protocols implemented in the UCSF Chimera software[9]. Grid location and dimensions were as in the CRDOCK protocol, but the spacing parameter was 0.3 Å. The default parameters for flexible docking and final minimization were maintained during docking calculations, and the 20 poses with lowest energy scores were clustered as the best CRDOCK docking solutions. AutoDock4.2 implements the Lamarckian genetic algorithm (LGA) which combines local search and a genetic algorithm[10]. All the active torsion angles of the ligand were allowed to rotate during docking, and the structure of the receptor was converted to pdbqt (AutoDock) format, conserving the partial charges, using AutoDock4 Tools program. The grid box was generated with 40 × 40 × 40 points at 0.375 Å resolution and the grid center was, again, the Nη2 atom of Arg271. The docking was performed with 250 runs, a population size of 300, 2.5×10^7 energy evaluations, and default values for the other docking parameters. The 25 solutions with lowest energy were selected. The fourth method, AutoDock-Vina (version 1.1.2), is based on a variety of stochastic global optimization

approaches, including genetic algorithms, particle swarm optimization and simulated annealing, between others, combined with several local optimization procedures[11]. The coordinates for the center of the grid were those used in the other approaches and grid dimensions were 16 × 16 × 16 Å. The exhaustiveness of the docking was increased to 16 binding modes and the 20 lowest-energy solutions were considered for further analysis.

References

1. Juanhuix, J. *et al*. Developments in optics and performance at BL13-XALOC, the macromolecular crystallography beamline at the Alba Synchrotron. *J. Synchrotron Radiat.* **21**, 679–689 (2014).
2. Baley, S. The CCP4 suite, programs for protein crystallography. *Acta Cryst. D***50,** 157-163, (1994).
3. Millán, C., Sammito, M. & Usón I. Macromolecular *ab initio* phasing enforcing secondary and tertiary structure. *IUCr J.* **2**, 95–105 (2015).
4. Vagin, A. & Teplyakov, A. Molecular replacement with MOLREP. *Acta Cryst.* D**66**, 22–25 (2010).
5. Emsley, P. & Cowtan, K. Coot: model-building tools for molecular graphics. *Acta cryst.* **D 60,** 2126–2132 (2004)
6. Adams, P. D. *et al*. PHENIX: a comprehensive Python-based system for macromolecular structure solution. *Acta Cryst.* **D66,** 213–221 (2010).
7. Cabrera, A. C. *et al*. CRDOCK: An ultrafast multipurpose protein-ligand docking tool. *J. Chem. Inf. Model* **52,** 2300–2309 (2012)Case, D.A. *et al.* AMBER 12, University of California, San Francisco (2012).
8. Pettersen, E. F. *et al*. UCSF Chimera–a visualization system for exploratory research and analysis. *J. Comput. Chem.* **25,** 1605–1612 (2004).
9. Morris, G. M. et al. AutoDock4 and AutoDockTools4: Automated docking with selective receptor flexibility. *J. Comput. Chem.* **30,** 2785–2791 (2009).
10. Trott, O. & Olson, A. J. AutoDock Vina: improving the speed and accuracy of docking with a new scoring function, efficient optimization, and multithreading. *J. Comput. Chem.* **31**, 455–461 (2010).
11. Bernardó, P., Mylonas, E., Petoukhov, M. V., Blackledge, M. & Svergun, D. I. Structural characterization of flexible proteins using small-angle X-ray scattering. *J. Amer. Chem. Soc.* **129,** 5656-5664 (2007).
12. Svergun, D., Barberato, C. & Koch, M. H. J. CRYSOL – A program to evaluate x-ray solution scattering of biological macromolecules from atomic coordinates. *J. Appl. Crystallogr.* **28,** 768–773 (1995).
13. Laskowski, R. A. & Swindells, M. B. LigPlot+: multiple ligand–protein interaction diagrams for drug discovery. *J. Chem. Inf. Model.* **51**, 2778–2786 (2011).
14. Meroueh, S.O. *et al*. Three dimensional structure of the bacterial cell wall peptidoglycan. *Proc. Natl. Acad. Sci. USA.* **103,** 4404-4409 (2006).
15. Salentin, S., Schreiber, S., Haupt, V. J., Adasme, M. F. & Schroeder, M. PLIP: fully automated protein-ligand interaction profiler. *Nucl. Acids Res*. **43** (W1), W443-W447 (2015).

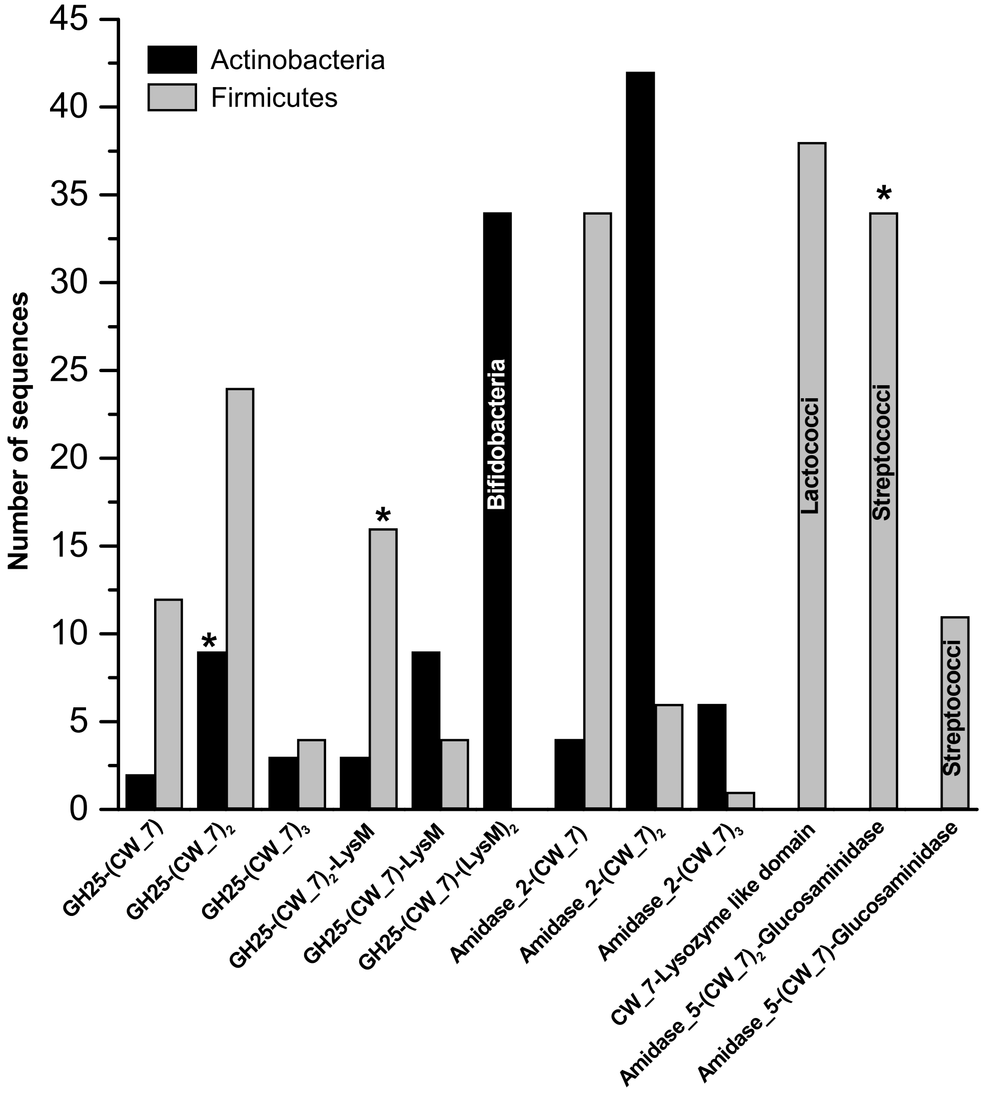

Supplementary Figure S1. Frequence of most populated architectures carrying CW_7 repeats in the Actinobacteria and Firmicutes phyla. Generally, repeats from Actinobacteria are three amino acids shorter than those from Firmicutes. However, repeats of GH25-(CW_7)$_2$-LysM and Amidase_5-(CW_7)$_2$-Glucosaminidase architectures are almost all short, and those of GH25-(CW_7)$_2$ long, independently of their origin (deviations from general rule are market with asterisks). Coexistence of long and short repeats only appears in Amidase_2-(CW_7)$_2$ sequences.

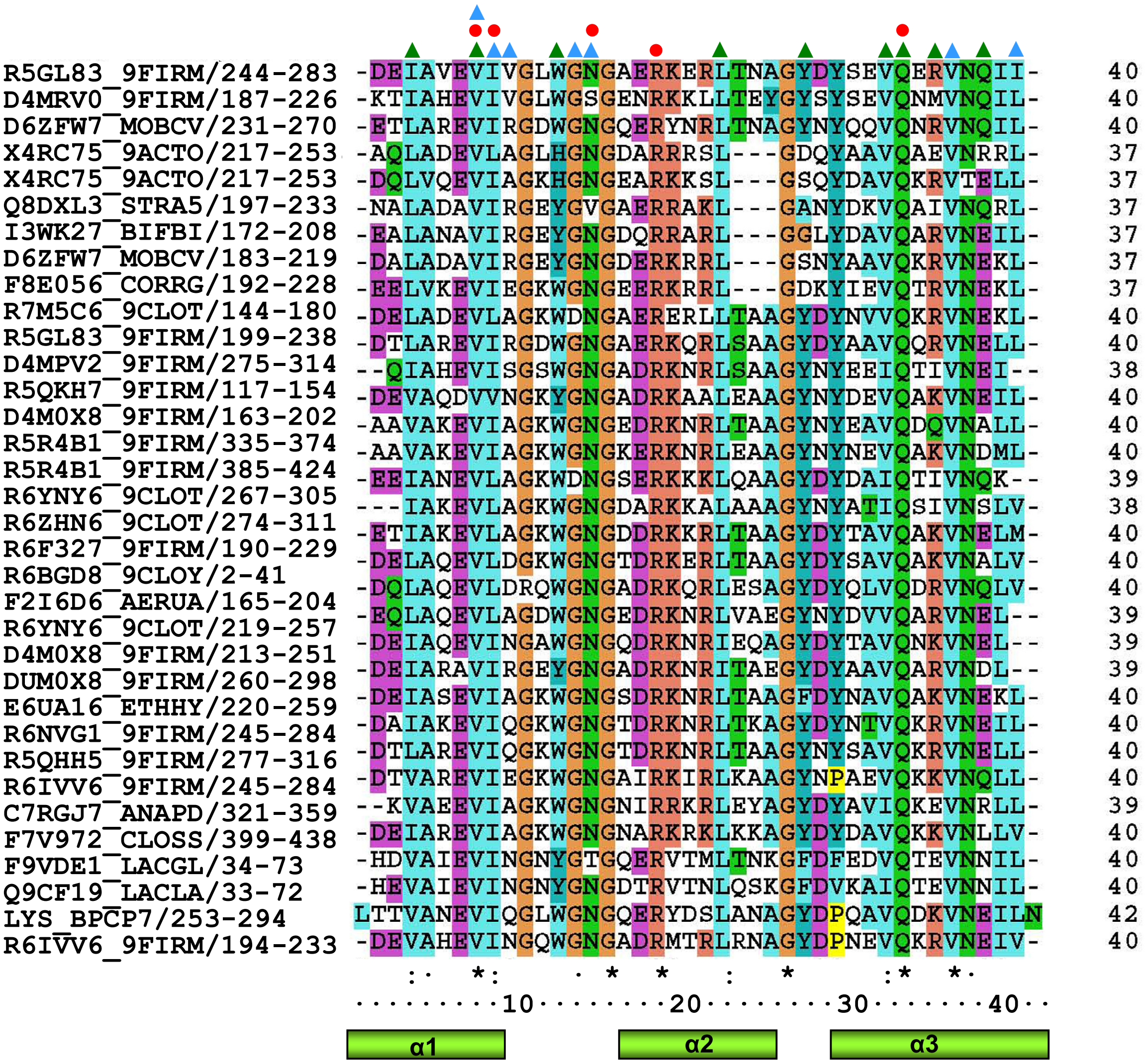

Supplementary Figure S2. Sequence conservation in the CW_7 motifs. Alignment of representative sequences of the CW_7 motif (Pfam PF08230; InterPro IPR013168). The UNIPROT accession code is shown. Asterisk, colons and dots indicate strictly conserved residues, conservative, and semi-conservative substitutions, respectively. Coloured mark-up is glycine (orange), proline (yellow), small hydrophobic (light-blue); tyrosine and histidine (dark blue); hydroxyl an neutral polar (green); acidic (magenta); and basic (red). Relevant residues for bundle stabilization are indicated by green triangles; and those involved in GMDP binding by red circles (hydrogen-bonds) or blue triangles (hydrophobic contacts). Green cylinders indicate α-helix positions in R2 crystal structure.

a

Amidase_2-(CW_7)$_2$

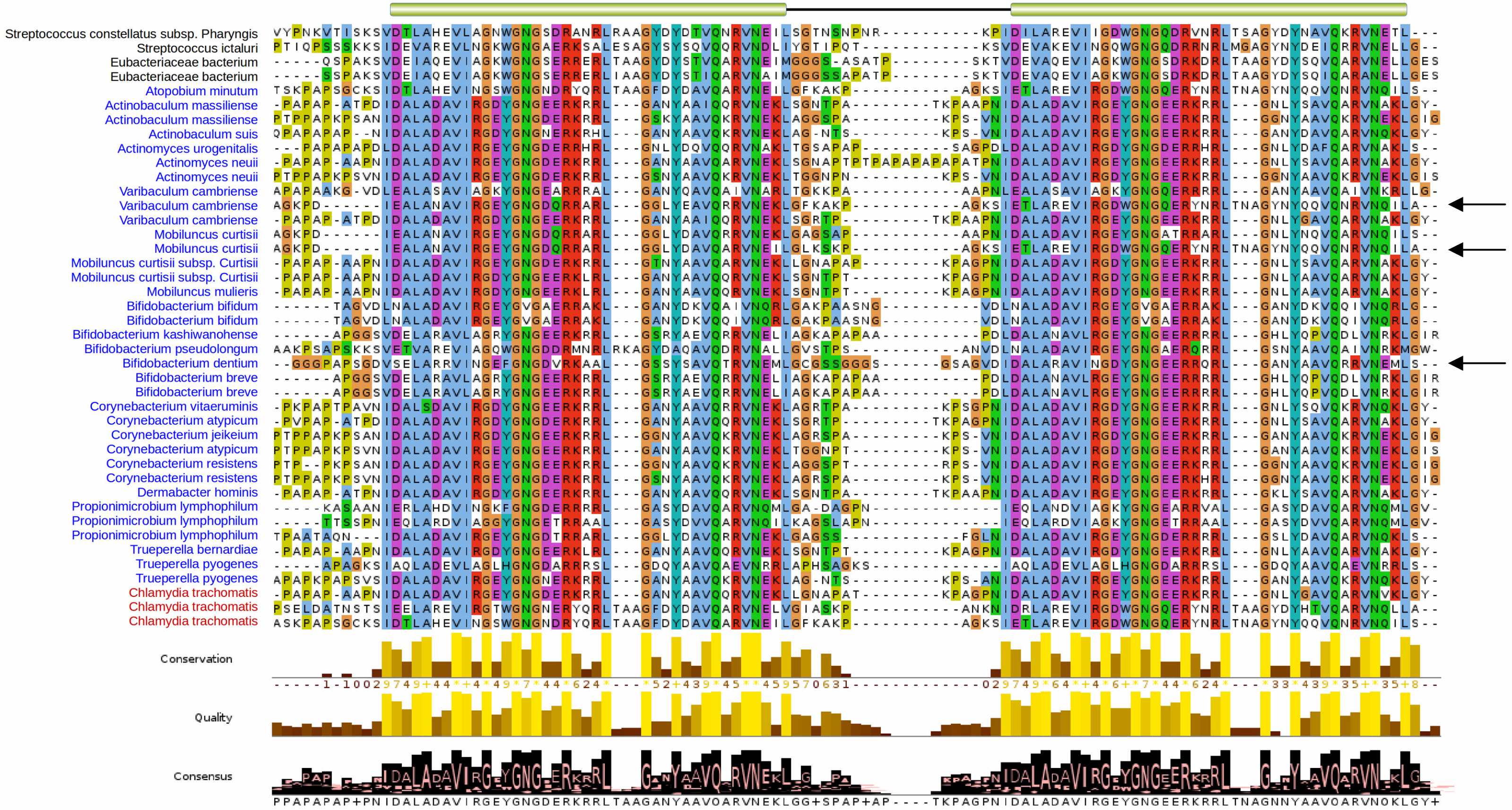

b

Amidase_2-(CW_7)$_3$

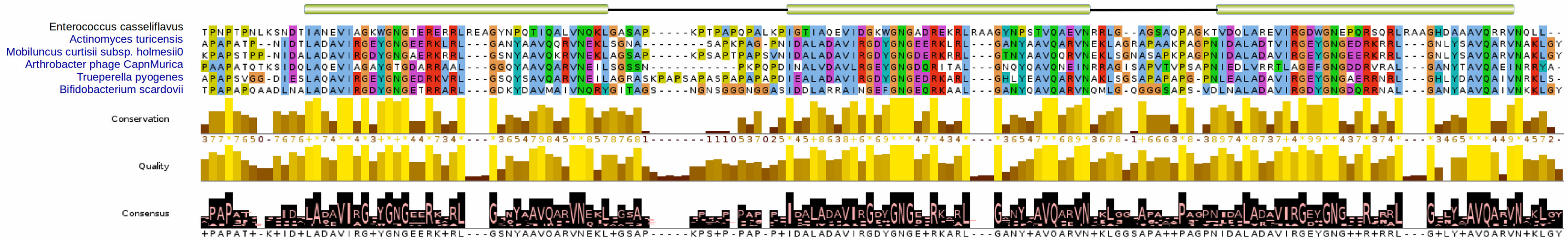

c GH25-(CW_7)$_2$

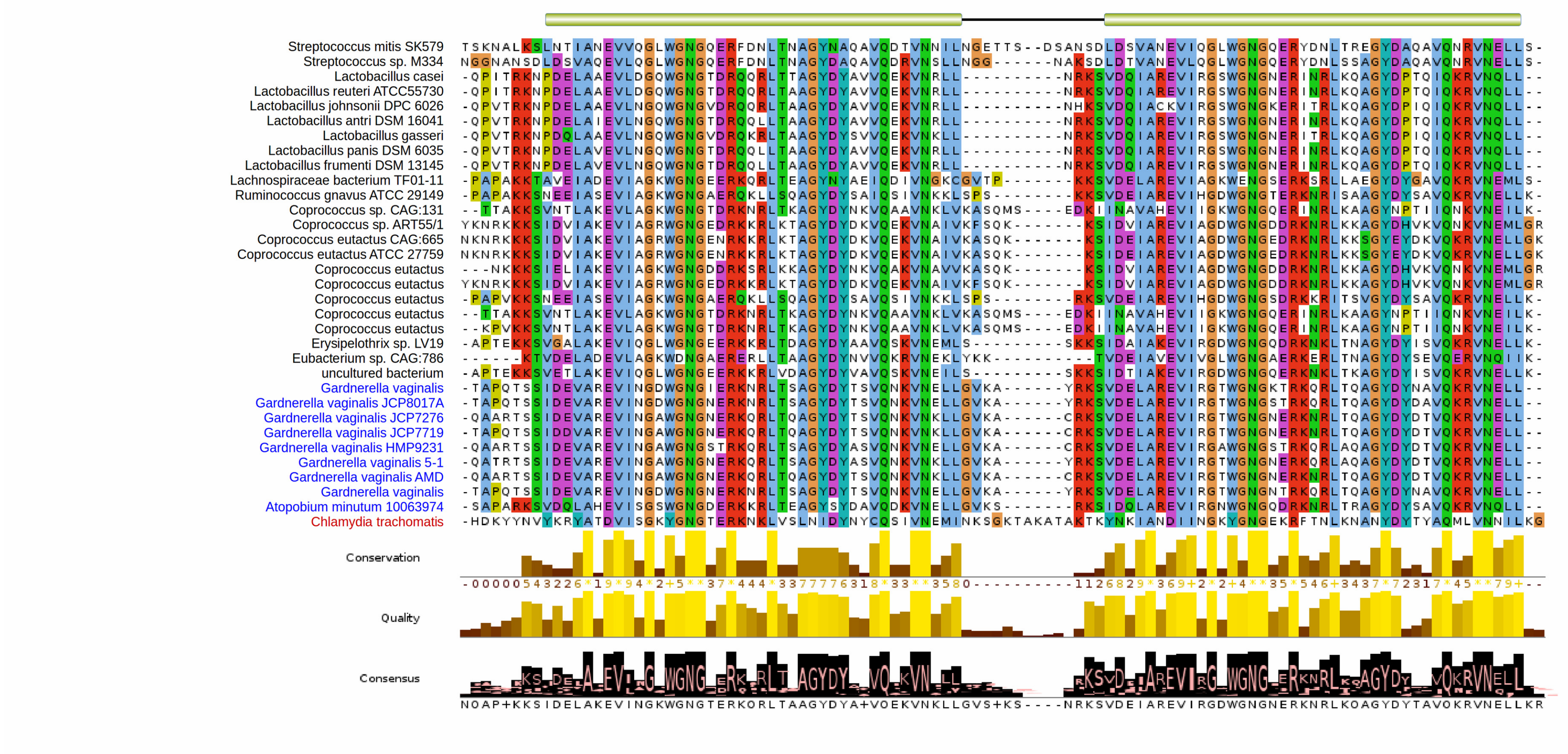

d GH25-(CW_7)$_3$

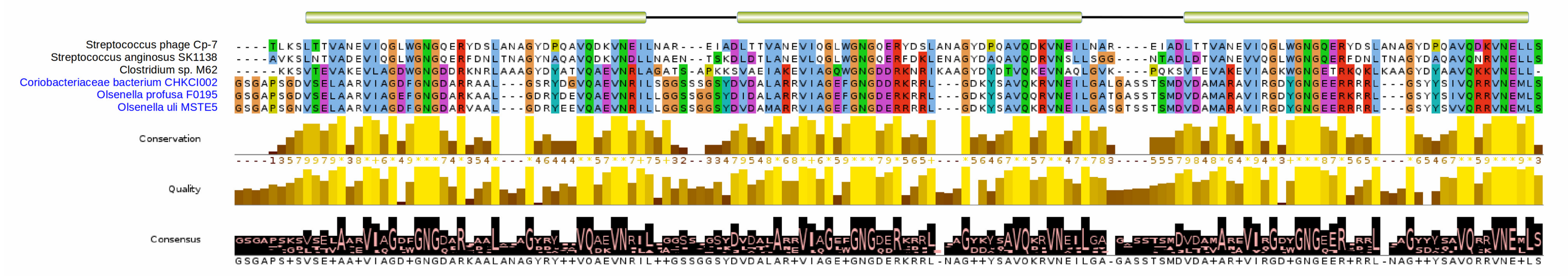

e

GH25-(CW_7)$_2$-LysM

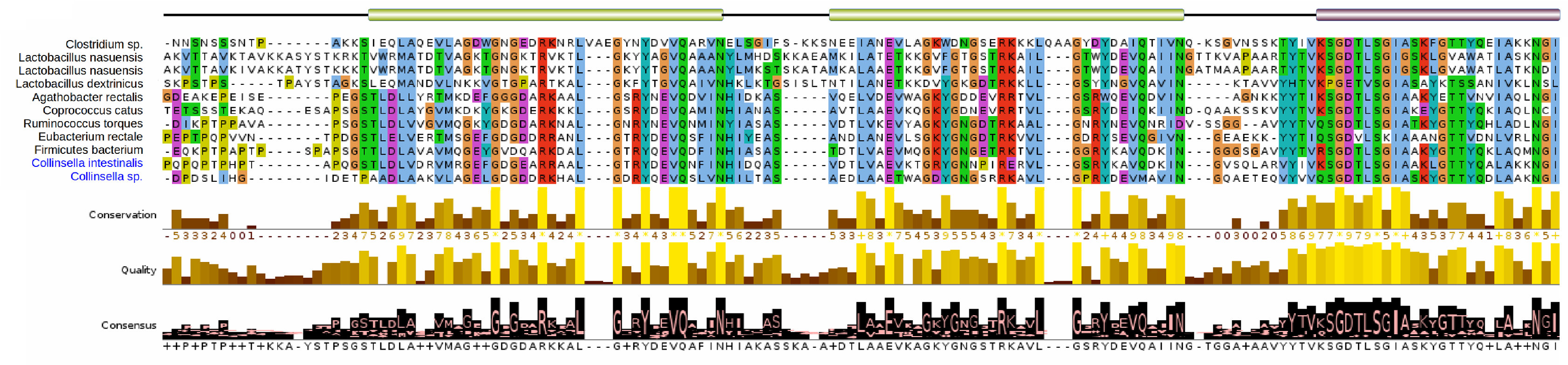

f

GH25-CW_7-(LysM)$_2$

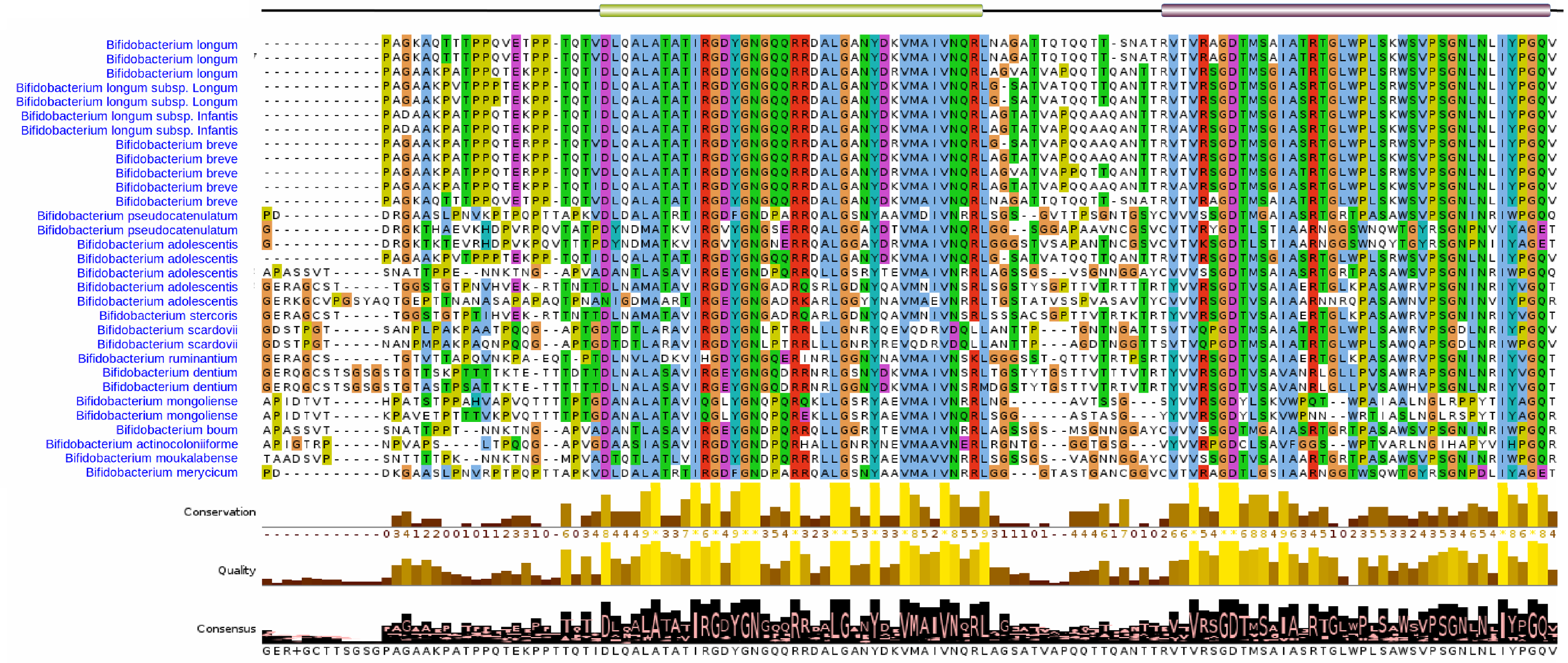

Supplementary Figure S3. Alignments of CW_7 repeats and interconnecting linkers of sequences comprised in architectures of (a) Amidase_2-$(CW\_7)_2$ (b) Amidase_2-$(CW\_7)_3$, (c) GH25-$(CW\_7)_2$ (d) GH25-$(CW\_7)_3$, (e) GH25-$(CW\_7)_2$-LysM and (f) GH25-(CW_7)-$LysM_2$. Coding bacteria are indicated in blue for Actinobacteria, black for Firmicutes and red for Clamydia. Changing patterns in amino acid distribution among repeats of the same sequence, repeats of different architectures, and inter-repeat connecting linkers are highlighted by colouring residues as in Supplementary Fig. S3. Linkers connecting CW_7 repeats in Amidase_2-$(CW\_7)_{2/3}$ sequences are rich in prolines and/or glycines and largely differ from those of GH25-$(CW\_7)_{2/3}$ sequences. Amidase_2-$(CW\_7)_3$ sequences in which short and long repeats coexist are indicated by arrows (panel a).

Cpl-7 wt

MVKKNDLFVDVASHQGYDISGILEEAGTTNTIIKVSESTSYLNPCLSAQVSQSNPIGFYHFAWFGGNEEEAEA
EARYFLDNVPTQVKYLVLDYEDHASASVQRNTTACLRFMQIIAEAGYTPIYYSYKPFTLDNVDYQQILAQFPN
SLWIAGYGLNDGTANFEYFPSMDGIRWWQYSSNPFDKNIVLLDDEKEDNINNENTLKSLTTVANEVIQGLWGN
GQERYDSLANAGYDPQAVQDKVNEILNAREIADLTTVANEVIQGLWGNGQERYDSLANAGYDPQAVQDKVNEI
LNAREIADLTTVANEVIQGLWGNGQERYDSLANAGYDPQAVQDKVNELLS

Cpl-7Δ1

MVKKNDLFVDVASHQGYDISGILEEAGTTNTIIKVSESTSYLNPCLSAQVSQSNPIGFYHFAWFGGNEEEAEA
EARYFLDNVPTQVKYLVLDYEDHASASVQRNTTACLRFMQIIAEAGYTPIYYSYKPFTLDNVDYQQILAQFPN
SLWIAGYGLNDGTANFEYFPSMDGIRWWQYSSNPFDKNIVLLDDEKEDNINNENTLKSLTTVANEVIQGLWGN
GQERYDSLANAGYDPQAVQDKVNEILNAREIADLTTVANEVIQGLWGNGQERYDSLANAGYDPQAVQDKVNEL
LS

Cpl-7Δ2

MVKKNDLFVDVASHQGYDISGILEEAGTTNTIIKVSESTSYLNPCLSAQVSQSNPIGFYHFAWFGGNEEEAEA
EARYFLDNVPTQVKYLVLDYEDHASASVQRNTTACLRFMQIIAEAGYTPIYYSYKPFTLDNVDYQQILAQFPN
SLWIAGYGLNDGTANFEYFPSMDGIRWWQYSSNPFDKNIVLLDDEKEDNINNENTLKSLTTVANEVIQGLWGN
GQERYDSLANAGYDPQAVQDKVNELLS

C-Cpl-7 wt

ENTLKSLTTVANEVIQGLWGNGQERYDSLANAGYDPQAVQDKVNEILNAREIADLTTVANEVIQGLWGNGQER
YDSLANAGYDPQAVQDKVNEILNAREIADLTTVANEVIQGLWGNGQERYDSLANAGYDPQAVQDKVNELLS

R_{1-2}

GEFNNENTLKSLTTVANEVIQGLWGNGQERYDSLANAGYDPQAVQDKVNEILNAREIADLTTVANEVIQGLWG
NGQERYDSLANAGYDPQAVQDKVNEILNAREIADLTTV

N-Cpl-7

MVKKNDLFVDVASHQGYDISGILEEAGTTNTIIKVSESTSYLNPCLSAQVSQSNPIGFYHFAWFGGNEEEAEA
EARYFLDNVPTQVKYLVLDYEDHASASVQRNTTACLRFMQIIAEAGYTPIYYSYKPFTLDNVDYQQILAQFPN
SLWIAGYGLNDGTANFEYFPSMDGIRWWQYSSNPFDKNIVLL

Supplementary Figure S4. Amino acid sequences of proteins used in this work. The one letter amino acid code is highlighted using the colour code of **Fig. 1** to identify the catalytic domain (green) and the three CW_7 repeats of the cell wall binding domain (R1: blue; R2: orange; and R3: yellow). Residues mutated to alanine in C-Cpl-7R, C-Cpl-7Q and C-Cpl-7I are highlighted in grey, red and cyan, respectively, in the C-Cpl-7 wt sequence. Those left at the beginning of the R_{1-2} fragment by the cut of LSLt-C-Cpl-7wt fusion protein with the TEV protease are highlighted in light blue.

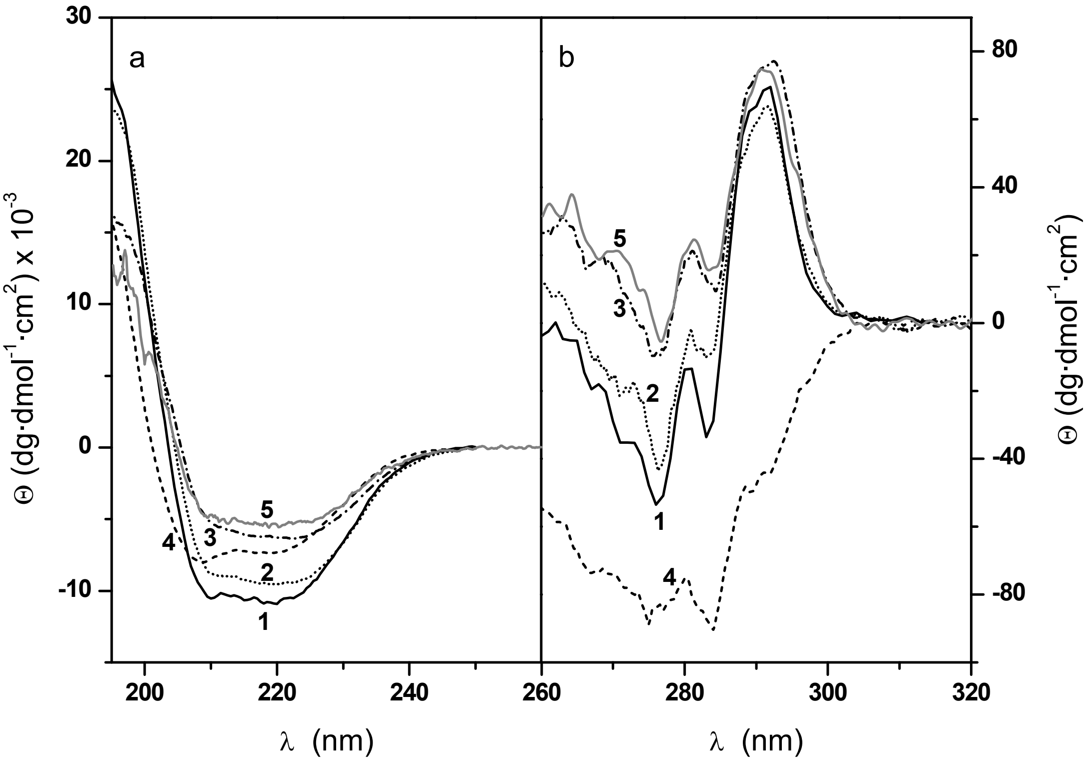

Supplementary Figure S5. **Comparision of the CD spectra of Cpl-7 and its separate domains with those of the CW_7 deletion mutants**. (**a**) Far- and (**b**) near-UV CD spectra of Cpl-7 wt (1), Cpl-7Δ1 (2), Cpl-7Δ2 (3), and C-Cpl-7 wt (4) and N-Cpl-7 (5) in PB buffer, pH 7.0, at 20 ºC. Average molar ellipticities per residue are weighted by the fractional contribution of respective sequences in the full-length protein Cpl-7.

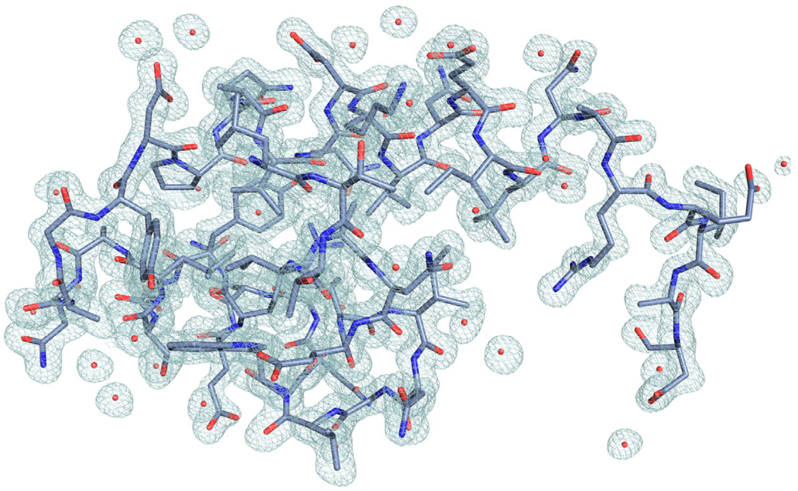

Supplementary Figure S6. 2Fo-Fc electron density map of the single repeat (R2) contoured at 1 σ in the unit-cell of R_{1-2} crystals. The protein is represented in sticks, waters as red spheres and the grid is depicted in pale blue.

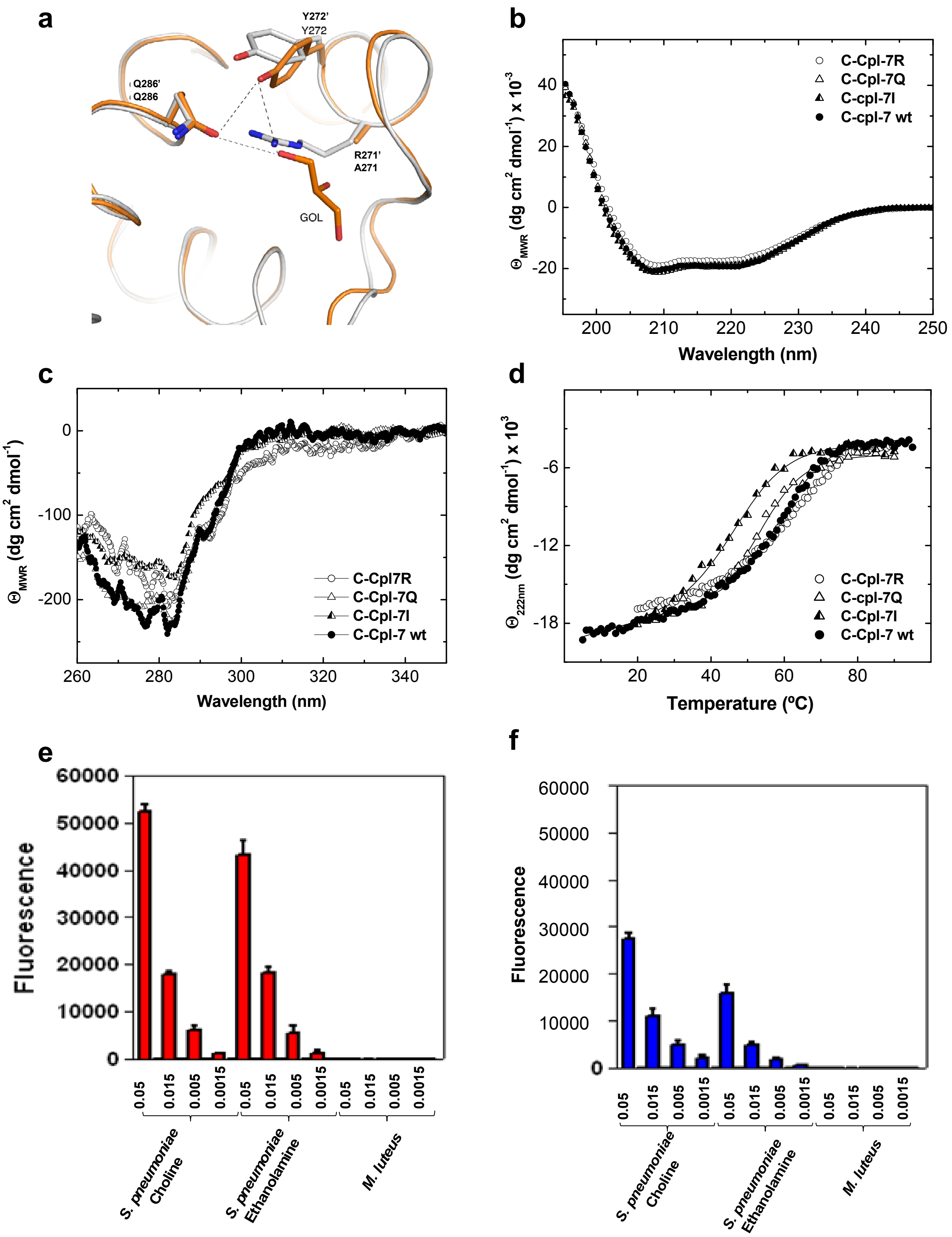

Supplementary Figure S7. **Effect of the triple arginine-to-alanine mutation on C-Cpl-7 structure and cell wall-binding activity**. (**a**) Superimposition of R2 structure from full-length C-Cpl-7R (orange) and the R_{1-2} fragment (white); residues affected by the mutation are in stick representation. Loss of the cation-π interaction between Tyr272 and Arg271 in C-Cpl-7R approached side chains of Tyr272 and Gln286 to hydrogen-bond distance, which probably explains the differences found in the near-CD spectra of panel (**c**). One molecule of glycerol (labelled as GOL) forms hydrogen bonds with both residues. (**b**) and (**c**) Far- and near-UV CD spectra of C-Cpl-7 wt and triple mutants in PB buffer at 20 ºC. (**d**) Thermal denaturation profiles of these constructs in PB. Solid traces are the theoretical fits of data to a two-state reversible denaturation model with T_m and ΔH_d values summarized in Supplementary Table S4. (**e**) and (**f**) Microarray binding assays of C-Cpl-7 wt and C-Cpl-7R, respectively, to purified cell walls of *S. pneumoniae* (grown in choline- or ethanolamine-containing media) or *Micrococcus luteus* (negative control) immobilized at four different doses (mg/ml). Substitution of choline by alanine has little or no effect on C-Cpl-7 binding.

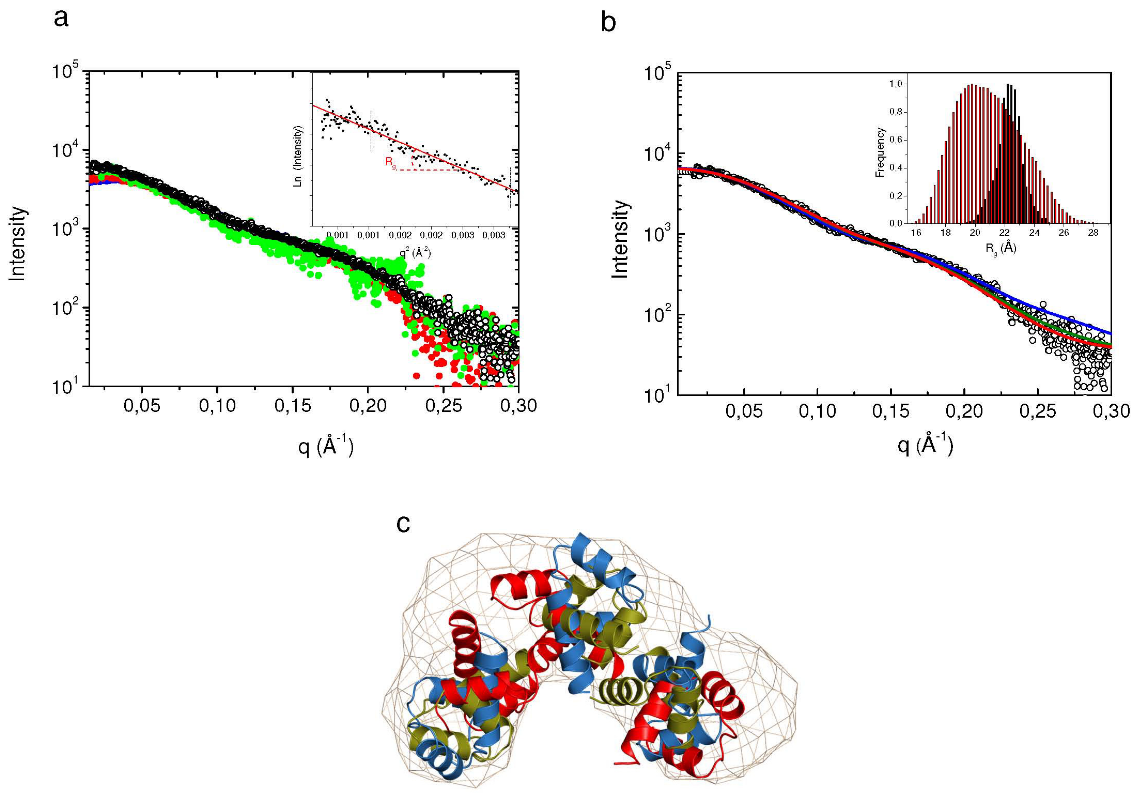

Supplementary Figure S8. SAXS profiles and 3D modelling of C-Cpl-7 wt in solution. (**a**) Extrapolation of C-Cpl-7 profiles to infinite dilution (black line) using the spectra recorded at 7.6, 3.8 and 1.9 mg/ml (blue, green and red dots, respectively). The inset shows the estimation of the radius of gyration (R_g) from the Guinier plot at infinite dilution. (**b**) Analysis of SAXS data using EOM[12]. The inset shows the broad R_g distribution of the starting pool of 10,000 random configurations (red bars) and the narrow distribution of the sub-ensemble of models selected by EOM (black bars). (**c**) Cartoon representation of a sub-ensemble of three C-Cpl-7 models selected by EOM superimposed with the *ab initio* envelope generated with DAMMIF (grey mesh). The theoretical spectra (blue, green and red traces) calculated with CRYSOL[13] from EOM models are compared with the experimental profile in panel (b).

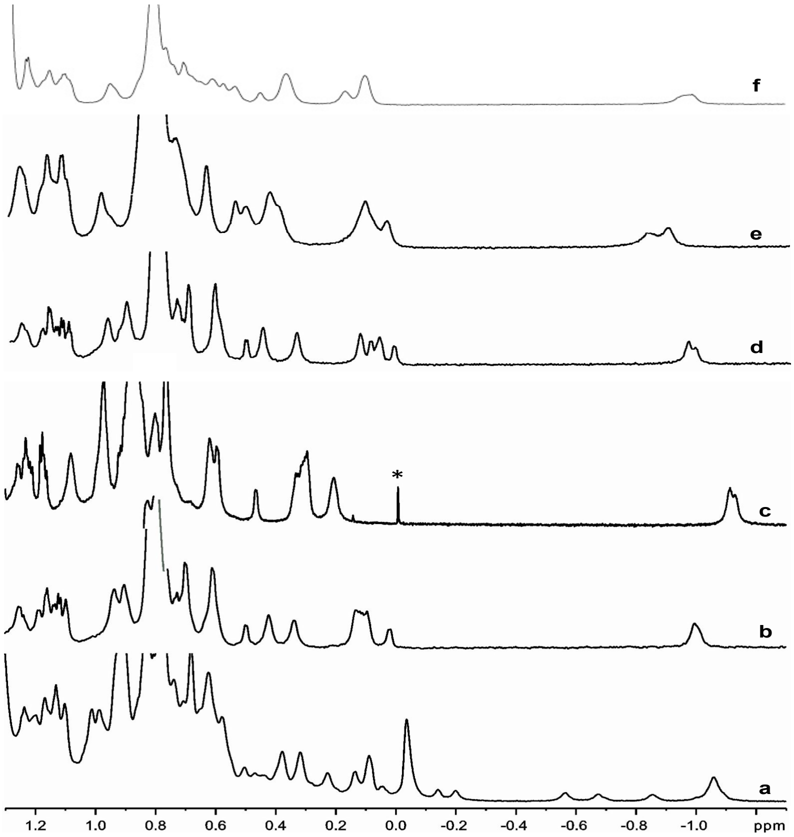

Supplementary Figure S9. **Comparison of the high field region (1.3 to -1.3 ppm) of the ^{1}H NMR spectra (800 MHz)** in PB-D_2O buffer of (**a**) Cpl-7 full length, (**b**) C-Cpl-7 wt, and (**c**) C-Cpl-7R, (**d**) C-Cpl-7, (**e**) C-Cpl-7I and (**f**) C-Cpl-7I in the presence of GMDP. The asterisk indicates the signal corresponding to the chemical shift reference (4,4-dimethyl-4-silapentane-1-sulfonic acid) added to the sample.

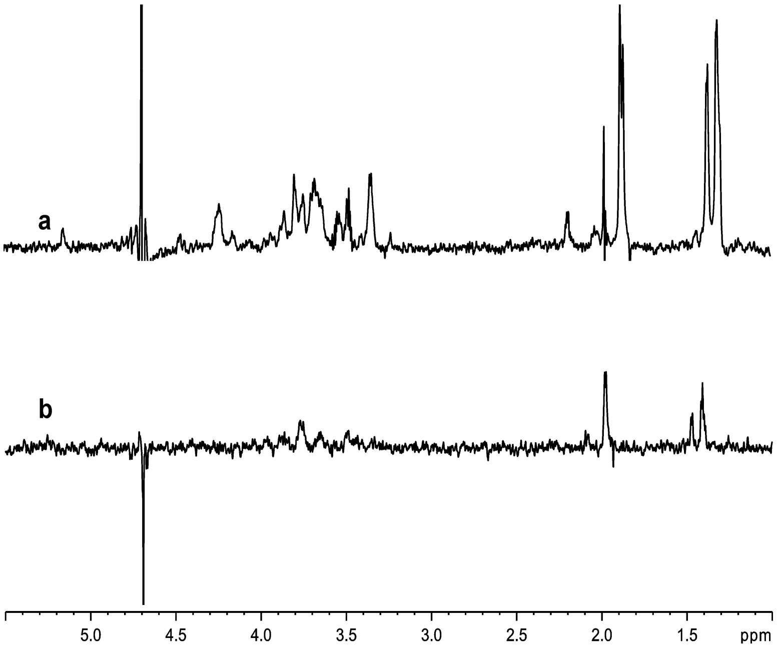

Supplementary Figure S10. **Exposure to high salt conditions impairs GMDP recognition by C-Cpl-7 wt.** The STD spectra (methyl irradiation) of GMDP in the presence of C-Cpl-7 wt became extremely weak on passing from (**a**) PB-D_2O buffer (I = 0.044; pD = 7.1) to (**b**) 0.25 M pyrophosphate, 1.5 M KCl (I = 3.3; pD = 8.6). NMR conditions were the same for both samples.

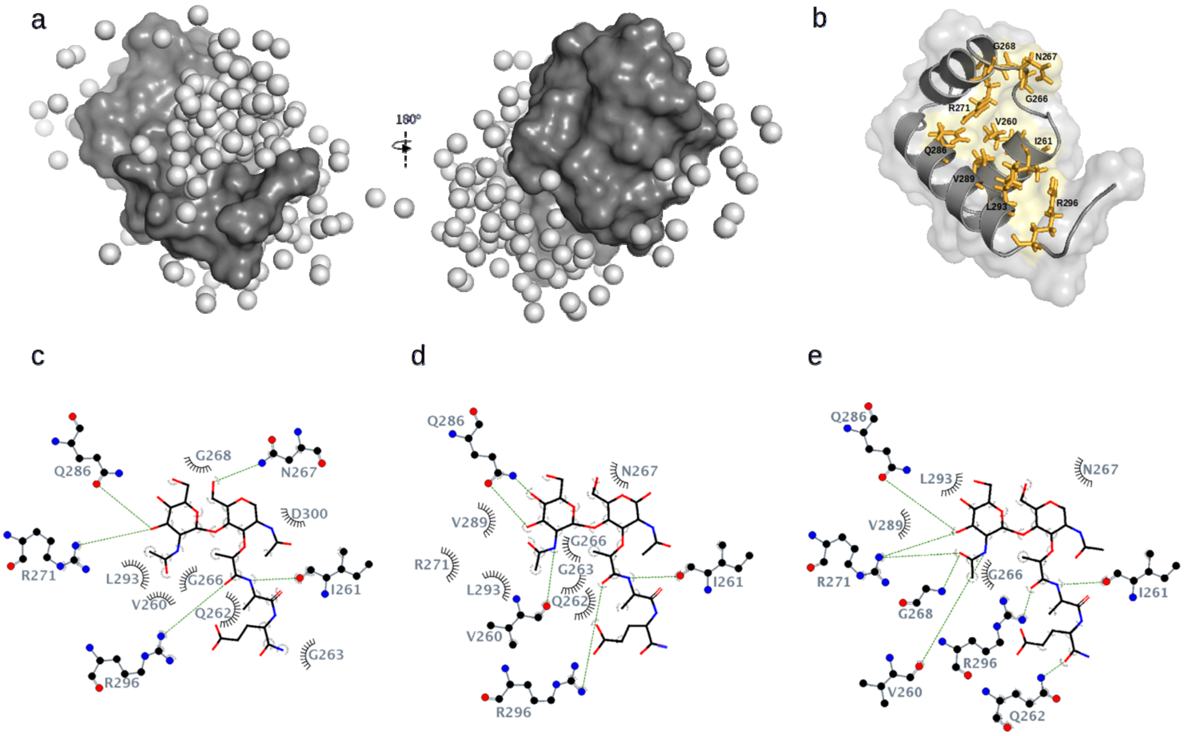

Supplementary Figure S11. GMDP docking into a CW_7 motif of Cpl-7. (**a**) Two different views of the blind docking performed with the AutoDock4.2 program. The receptor surface is coloured in grey and the relative positions of the MurNAc and GlcNAc units of GMDP are indicated by spheres. (**b**) View of the GMDP binding surface. Relevant residues for ligand recognition inferred from the best docking models generated with AutoDock Vina, CRDOCK, DOCK6 and AutoDock4 are shown in stick representation (R2 sequence numbering). LigPlot[14] representations of contacts between GMDP and the R2 repeat of Cpl-7 wt in the best docking models of (**c**) AutoDock Vina, (**d**) CRDOCK and (**e**) DOCK6. Dashed lines indicate the hydrogen bonds and the black arcs van der Waals interactions.

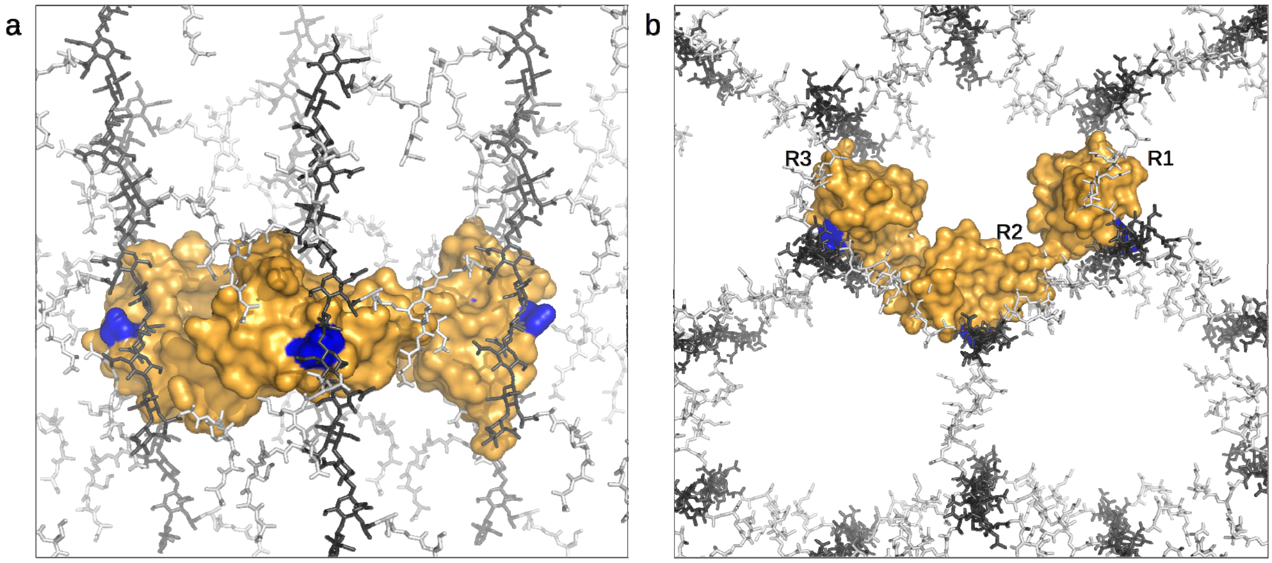

Supplementary Figure S12. Views from the side (a) and above (b) of the SAXS based model of C-Cpl-7 superimposed into a pore of the NMR-based structure of the cell wall proposed by Meroueh *et al.*[15]. The glycan strands (black) and the peptide stems (grey) are shown in stick representation. The C-Cpl-7 domain (surface representation) is shown in orange with the position of the GMDP binding comprised in each repeat highlighted in blue. Their dispositions are compatible with a 1:3 domain-to-ligand stoichiometry involving three different glycan chains.

Supplementary Tables

Supplementary Table S1 | Sedimentation coefficients ($s_{20,w}$), Stokes' radii (Rs) and frictional coefficients (f/f_0) of Cpl-7 and the repeat deletion mutants measured by sedimentation velocity at 20 ºC.

Protein	$s_{20,w}$ (S)	R_s (Å)	f/f_0
Cpl-7 wt	2.73	34.1	1.51
Cpl-7Δ1	2.53	31.8	1.48
Cpl-7Δ2	2.42	27.9	1.37

**Supplementary Table S2 | ^{1}H chemical shift data (ppm) of D-GlcNAc-(β 1-4)-D-MurNAc-L-Ala-D-isoGln (GMDP) at 25 ºC in PB-D_2O**		
Proton	Chemical shift	
GlcNAc-1	4.47	
GlcNAc-2	3.68	
GlcNAc-3	3.49	
GlcNAc-4	3.35	
GlcNAc-5	3.36	
GlcNAc-6	3.70	
GlcNAc-6'	3.87	
GlcNAc-(CH_3)	1.98	
GlcNAc-(NH)	8.34	
	α-anomer	β-anomer
MurNAc-1	5.16	4.57
MurNAc-2	3.77	3.65
MurNAc-3	3.79	3.54
MurNAc-4	3.69	3.79
MurNAc-5	n.a.	3.41
MurNAc-6	3.65	3.62
MurNAc-6'	3.75	3.82
MurNAc-(CH_3)	1.89	1.87
MurNAc-(NH)	7.89	7.67
D-Lac-α	4.49	4.36
D-Lac-β	1.32	1.30
L-Ala-NH	8.36	8.24
L-Ala-α	4.24	4.23
L-Ala-β	1.38	1.38
D-iGln-NH	8.49	8.54
D-isoGln-α	4.26	4.28
d-isoGln-NH2	7.61, 7.04	7.66, 7.07
d-isoGln-β	2.04	2.04
d-isoGln-β'	1.86	1.87
D-isoGln-γ,γ'	2.24	2.24

Supplementary Table S3 | Hydrogen bonds and hydrophobic contacts established between GMDP and the R2 repeat of C-Cpl-7 in the GMDP/R2 complex models generated by four different docking methods.[1]							
**Ligand unit**	**Residue**	**Distance H-A (Å)**	**Distance D-A (Å)**	**Angle (degrees)**	**Protein donor**	**Atoms[2]**	
			**AutoDock4.2 Solution**				
	**Hydrogen bonds**					**Donor[3]**	**Acceptor[3]**
GlcNAc	Val260	2.6	3.5	171.0	No	**N2**	CO
	Arg271	1.9	2.8	154.9	Yes	Nη2	**O2N**
	Arg271	2.7	3.0	113.3	Yes	Nη2	**O3**
	Gln286	2.5	3.5	173.3	Yes	Nε2	**O4**
	Gln286	2.4	3.2	150.3	No	**O3**	Oε1
MurNAc	Asn267	2.3	3.3	170.3	No	**O6**	Oδ1
L-Ala	Ile261	2.1	2.4	131.7	No	**NH**	CO
	**Salt bridge**						
D-IsoGln	Arg296	1.7	2.7	171.4	–	Nη1	**Oε1**
	**Hydrophobic contacts**						
GlcNAc	Gly266					**C2N**	Cα
	Val289					**C3**	C2
MurNAc	Arg296					**Cβ (lactyl)**	Cδ
L-Ala	Gln262					**Cβ**	Cα
	Ile261					**Cβ**	Cγ2
D-isoGln	Arg296					**Cγ**	Cδ
			**AutoDock Vina Solution**				
	**Hydrogen bonds**					**Donor**	**Acceptor**
GlcNAc	Arg271	2.7	3.1	104.1	Yes	Nη2	**O3**
	Gln286	3.4	3.8	108.4	No	**O3**	Oε1
MurNAc	Asn267	2.6	2.9	106.1	Yes	Nδ2	**O6**
	Arg296	2.5	3.2	138.7	Yes	Nη1	**CO (lactyl)**
L-Ala	Ile261	2.1	3.0	147.9	No	**NH**	CO
	**Hydrophobic contacts**						
GlcNAc	Gly266					**C2N**	Cα
	Leu293					**C4**	Cδ2
	Val260					**C2N**	Cγ2
MurNAc	Asp300					**Cβ (lactyl)**	Cβ
L-Ala	Gln262					**Cβ**	Cβ
	Ile261					**Cβ**	Cγ2
D-IsoGln	Gly263					**Cα**	Cα
			**CRDOCK Solution**				
	**Hydrogen bonds**					**Donor**	**Acceptor**
GlcNAc	Val260	2.2	3.1	167.5	No	**N2**	CO
	Gln286	2.9	3.3	111.4	No	**O3**	Oη2
	Gln286	2.3	3.3	170.1	Yes	NH	**O4**
MurNAc	Arg296	2.1	3.0	148.7	Yes	Nη1	**CO (lactyl)**
L-Ala	Ile261	2.1	2.9	137.1	No	**NH**	CO
	**Hydrophobic contacts**						

GlcNAc	Val260					**C2N**	Cγ1
	Arg271					**C2N**	Cγ
	Gly266					**C2N**	Cα
	Val289					**C3**	Cγ2
	Leu293					**Cβ (lactyl)**	Cδ2
MurNAc	Asn267					**C6**	Cβ
	Ile261					**Cβ (lactyl)**	Cγ2
L-Ala	Gln262					**Cβ**	Cγ
	Gly263					**Cβ**	Cα
			**Dock6.3 Solution**				
	**Hydrogen bonds**					**Donor**	**Acceptor**
GlcNAc	Val260	3.0	3.9	151.2	No	**N2**	CO
	Gly268	3.1	4.0	160.6	Yes	NH	**O2N**
	Arg271	2.2	3.1	151.5	Yes	Nη1	**O2N**
	Gln286	3.4	4.0	119.6	No	**O3**	Oε1
	Arg271	2.2	3.3	131.3	Yes	Nη1	**O3**
MurNAc	Arg296	2.3	3.0	131.8	Yes	Nη1	**CO (lactyl)**
L-Ala	Ile261	2.7	3.3	128.5	No	**NH**	CO
D-isoGln	Gln262	3.2	4.0	142.1	Yes	Nε2	**CO**
	**Hydrophobic contacts**						
GlcNAc	Gly266					**C2N**	Cα
	Leu293					**C4**	Cδ2
	Val289					**C3**	Cγ2
MurNAc	Asn267					**C6**	Cβ
	Arg296					**Cβ (lactyl)**	Cδ
L-Ala	Ile261					**Cβ**	Cγ2

[1]The analysis was performed with the PLIP server[16] and the Ligplot+ program[14] .
[2]GMDP atoms are shown in bold.
[3]Donor and acceptor apply only to hydrogen bonds.

Supplementary Table S4 | Thermal stability and hydrodynamic parameters at 20 ºC of C-Cpl-7 wt and its triple mutants

Protein	T_m (ºC)	$\triangle H_d$ (kcal/mol)	$s_{20,w}$ (S)	R_s (Å)	f/f_0
C-Cpl-7 wt	60.6 ± 0.1	33.8 ± 0.5	1.6	24.5	1.46
C-Cpl-7R	65.4 ± 0.2	31.1 ± 0.7	1.5	25.3	1.54
C-Cpl-7Q	54.4 ± 0.5	35 ± 2	1.6	24.0	1.42
C-Cpl-7I	48.8 ± 0.9	29 ± 1	1.5	25.0	1.49
